# Dose-Related Estrogen Effects on Gene Expression in Fetal Mouse Prostate Mesenchymal Cells

**DOI:** 10.1371/journal.pone.0048311

**Published:** 2012-10-29

**Authors:** Julia A. Taylor, Catherine A. Richter, Atsuko Suzuki, Hajime Watanabe, Taisen Iguchi, Kathryn R. Coser, Toshihiro Shioda, Frederick S. vom Saal

**Affiliations:** 1 Division of Biological Sciences, University of Missouri, Columbia, Missouri, United States of America; 2 Okazaki Institute for Integrative Bioscience, National Institute for Basic Biology, National Institutes of Natural Sciences, and Department of Basic Biology, Graduate University for Advanced Studies (SOKENDAI), Aichi, Japan; 3 Molecular Profiling Laboratory, Massachusetts General Hospital Cancer Center and Harvard Medical School, Charlestown, Massachusetts, United States of America; II Università di Napoli, Italy

## Abstract

Developmental exposure of mouse fetuses to estrogens results in dose-dependent permanent effects on prostate morphology and function. Fetal prostatic mesenchyme cells express estrogen receptor alpha (ERα) and androgen receptors and convert stimuli from circulating estrogens and androgens into paracrine signaling to regulate epithelial cell proliferation and differentiation. To obtain mechanistic insight into the role of different doses of estradiol (E2) in regulating mesenchymal cells, we examined E2-induced transcriptomal changes in primary cultures of fetal mouse prostate mesenchymal cells. Urogenital sinus mesenchyme cells were obtained from male mouse fetuses at gestation day 17 and exposed to 10 pM, 100 pM or 100 nM E2 in the presence of a physiological concentration of dihydrotestosterone (0.69 nM) for four days. Gene ontology studies suggested that low doses of E2 (10 pM and 100 pM) induce genes involved in morphological tissue development and sterol biosynthesis but suppress genes involved in growth factor signaling. Genes involved in cell adhesion were enriched among both up-regulated and down-regulated genes. Genes showing inverted-U-shape dose responses (enhanced by E2 at 10 pM E2 but suppressed at 100 pM) were enriched in the glycolytic pathway. At the highest dose (100 nM), E2 induced genes enriched for cell adhesion, steroid hormone signaling and metabolism, cytokines and their receptors, cell-to-cell communication, Wnt signaling, and TGF- β signaling. These results suggest that prostate mesenchymal cells may regulate epithelial cells through direct cell contacts when estrogen level is low whereas secreted growth factors and cytokines might play significant roles when estrogen level is high.

## Introduction

The mouse prostate begins to differentiate from the urogenital sinus (UGS) at gestation day 17, soon after the onset of testosterone secretion by the fetal testes [1]–[2]. Prostate duct development is initiated by mesenchymal influences and results in the formation of epithelial cell outgrowths, or epithelial buds. This event is dependent on mesenchymal conversion of testosterone to 5α-dihydrotestosterone (DHT), which is a higher-affinity androgen receptor (AR) ligand [1], [3]. Androgen receptor gene (Ar) expression in prostatic mesenchyme is required for the continued normal growth and branching morphogenesis of epithelial ducts [4], [5]. Although differentiation of the prostate is androgen-dependent, there is now considerable evidence that estrogens act to modulate the activity of androgen in regulating prostate development. During development, the mouse and rat UGS mesenchyme expresses both Ar and estrogen receptor- α (Esr1). In contrast, epithelial cells exhibit little androgen binding at this time, and Ar expression in UGS epithelium is not required for differentiation [6], [7], [8]. Since the growth of epithelial cells requires signals from the UGS mesenchyme [9], and since fetal UGS epithelial cells do not express estrogen receptors (ER) [8], [10], proliferative responses of the epithelial compartment to estrogens have been presumed to be driven by stimuli from mesenchymal cells.

We and others have shown that prenatal exposure of male mouse fetuses to estradiol-17β (E2), estrogenic drugs such as diethylstilbestrol (DES) and ethinylestradiol, or industrial estrogenic chemicals such as bisphenol A (BPA), induce an increase in the number of developing prostatic glands and an increase in prostate gland size during fetal life due to basal epithelial cell hyperplasia [11], [12], [13]; there is also a permanent increase in prostatic AR [11]. However, effects of prenatal estrogen exposure do not follow a monotonic dose response [14], and effects on the developing prostate at high and low concentrations may be very different [11], [12], [13]. We recently reported that the exposure of primary culture fetal mouse prostate mesenchyme cells to E2 enhanced expression of both Ar and Esr1 [14] in a non-monotonic manner. In the present study, we sought to identify other estrogen-regulated genes in mesenchymal cells and to compare effects of low (physiological) and high (pharmacological) concentrations of E2 on gene expression while the concentration of DHT was held constant. We show here that gene expression is dose-dependent but that expression profiles differ at low and high doses.

## Materials And Methods

### Ethics Statement

All animal procedures were approved by the University of Missouri Animal Care and Use Committee (protocol number: 6489) and conformed to the Guide for the Care and Use of Laboratory Animals of the National Institutes of Health. The program is fully accredited by the Association for Assessment & Accreditation of Laboratory Animal Care, International (AAALAC).

### Animals

CD-1 mice were purchased from Charles River Laboratories (Wilmington, MA) and maintained as an outbred stock at the University of Missouri. Animals were housed on corncob bedding in standard (11.5×7.5×5″) polypropylene cages. Water was purified by reverse osmosis and carbon filtration and provided in glass bottles *ad libitum*. Pregnant and lactating females were fed Purina 5008 chow, and otherwise were maintained on Purina 5001 chow. Rooms were maintained at 25±2°C under a 12∶12 L∶D cycle.

### Tissue collection, primary cell culture, and dosing

Timed-pregnant females were killed on gestation day (GD) 17 (mating = GD 0) by CO_2_ asphyxiation, and fetuses were removed from the uterine horns. The bladder and UGS were removed from male fetuses as previously described [8], [11], and the prostatic region of the UGS was separated from the bladder at the bladder neck and the lower UGS just below the ejaculatory ducts. UGS tissue was disrupted by collagenase treatment as described [14]. Epithelial and mesenchymal cells in the suspension were separated by gravity, since the epithelial cells settle and the mesenchymal cells remain suspended. The composition of the two cell type fractions was confirmed by immunofluorescence staining of cytokeratins with mouse anti-pan-cytokeratin clone PCK-26 fluorescein isothiocyanate conjugate (Sigma), and co-staining with the mesenchymal cell marker vimentin with goat anti-vimentin (Sigma) and rabbit anti-goat Cy3 conjugate (Sigma) ([15], data not shown). For these studies, epithelial cells were discarded and the collected mesenchymal cells were cultured at 37°C under 5% CO_2_ in RPMI-1640 medium without phenol red (Gibco, Grand Island, NY), supplemented with 2 mM L-glutamine, 100 units penicillin G sodium/ml, 100 mg streptomycin sulfate/ml, and 0.25 mg fungizone/ml. 10% fetal bovine serum (FBS) (U.S. Bio-Technologies, Parkerford, PA) was added to this initial growth medium and was not stripped of endogenous steroid hormones. Cells were grown to 95% confluence (approximately 3–5 days), and then passaged by digestion with 0.05% trypsin in 0.53 mM EDTA (Gibco) for 5 min at room temperature.

First passage cells were used in these experiments and were seeded at 3.2×10^5^ cells/well in 35 mm dishes. Cells were seeded in complete RPMI medium with endogenous hormones removed by substituting 5% (v/v) charcoal-stripped FBS and 5% (v/v) charcoal-stripped horse serum (Sigma, St. Louis, MO) for the 10% whole FBS, and further supplementing with ITS supplement (Cambrex, Walkersville, MD), for final concentrations of 10 µg insulin/ml, 10 µg transferrin/ml, and 10 ng selenium/ml. This medium was further supplemented with 690 pM DHT (200 pg/ml). Cells were treated with DHT rather than testosterone for two reasons. First, we wanted to control E2 exposure, since the developing prostate expresses aromatase [16] and unlike testosterone, DHT is not aromatized to E2 [5]. Second, we aimed to control the androgen concentration due to the potential for these compounds to alter 5α-reductase activity [17]. Cells were maintained in estrogen-free medium for three days, with one medium change, before the start of estrogen treatment, and then treated with either low doses (10 pM and 100 pM) or a high dose (100 nM) of E2 [14], selected based on our previous work (14). Negative controls were treated with the treatment vehicle, 0.05% ethanol. Cells were treated for four days with daily medium changes, with three replicate samples per treatment. At the end of the treatment period the cells were washed once with PBS, and immediately lysed on ice in Trizol (Invitrogen, Carlsbad, CA).

### Microarray Analysis

Total RNA was isolated from the Trizol lysate and purified with the RNeasy Mini kit (Qiagen, Valencia, CA) according to the manufacturers' instructions, and RNA quality was checked on an Agilent Bioanalyzer (Agilent, Palo Alto, CA). The transcriptomal profiles were determined using Affymetrix mouse ST 1.0 or 430A microarrays. Scanned image data were converted into numerical tables using Affymetrix GeneChip Operating Software and Gene Expression Console. Data analysis and mining, including gene ontology enrichment analysis, were performed using GeneSifter server (Giospiza Inc., Seattle, WA) and Partek Genomics Suite (Partek Inc., St. Louis, MO). Microarray data were deposited in NCBI Gene Expression Omnibus (accession numbers GSE16854 and GSE36630).

### Quantitative PCR (qPCR) measurement of gene expression

To confirm the relative changes in gene expression induced by estradiol, we used a real-time quantitative reverse transcriptase-polymerase chain reaction (qPCR) approach for selected transcripts [18]. These data were previously reported elsewhere, compared with results for BPA treatment [19]; that article is attached here in Supporting Information. Fetal mouse UGS mesenchyme cells were treated *in vitro* for four days as described with either 100 nM 17β-estradiol or the ethanol vehicle (0.05%) alone; treatments were performed in triplicate wells within each experiment, and analyses were conducted on RNA preparations from three independent experiments. Total RNA was isolated with the RNAqueous kit (Ambion, Austin, TX) according to the manufacturer's instructions, and quantified by absorbance at 260 nm. Expression of specific mRNAs were measured by one-step real time Rt-PCR as described [20] using the TaqMan EZ RT-PCR kit (PE Applied Biosystems, Foster City, CA) on the ABI Prism 7700 Sequence Detection System (PE Applied Biosystems). Assays for each mRNA were carried out in duplicate. The primer/probe set for Ar was designed using Primer Express software (PE Applied Biosystems), as described [14]. Ar primers were synthesized by Invitrogen, and the Ar probe was synthesized by Applied Biosystems. The concentrations of Mn^2+^, probe and primers were optimized for the primer/probe set. Other analyses were performed using validated ABI Taqman Gene Expression assays (Applied Biosystems). Assays for each mRNA were carried out in duplicate. ABI Taqman Gene Expression assays used for specific transcripts were: Mm00433149_m1 (Esr1), Mm00432087_m1 (Bmp4), Mm00500361_m1 (Capn6), Mm00484157_m1 (Cyp7b1), Mm00840104_m1 (Sfrp4) and Mm00449036_m1 (Thbs2). These primers spanned *Esr1* exons 3–4, Bmp4 exons 2–3, Capn6 exons 2–3, Cyp7b1 exons 4–5, Sfrp4 exons 4–5 and Thbs2 exons 1–2.

The relative concentrations of specific mRNAs in each sample were normalized to total RNA per well, as described [20], [21]. Normalization to total RNA allowed for comparisons between independent experiments and provided a conservative estimate of relative amounts of mRNA. Differences between control and estradiol -treated cells were evaluated using the ANOVA GLM procedure in SAS. Comparisons of mean reciprocals for each dose relative to controls were made using the LS Means Test in SAS. The criterion for statistical significance was P≤0.05 (two-tailed).

## Results

### Microarray analysis of effects of low (10 pM and 100 pM) concentrations of E2 on gene expression in fetal mouse prostate cells

Exposure of the primary culture mouse prostate mesenchymal cells significantly affected expression of 628 genes (ANOVA, p<0.01, unadjusted). Benjamini-Hochberg correction of multiple testing eliminated these effects, reflecting the relatively low statistical power of the present analysis due to the limited numbers of samples in each group.

**Figure 1:**
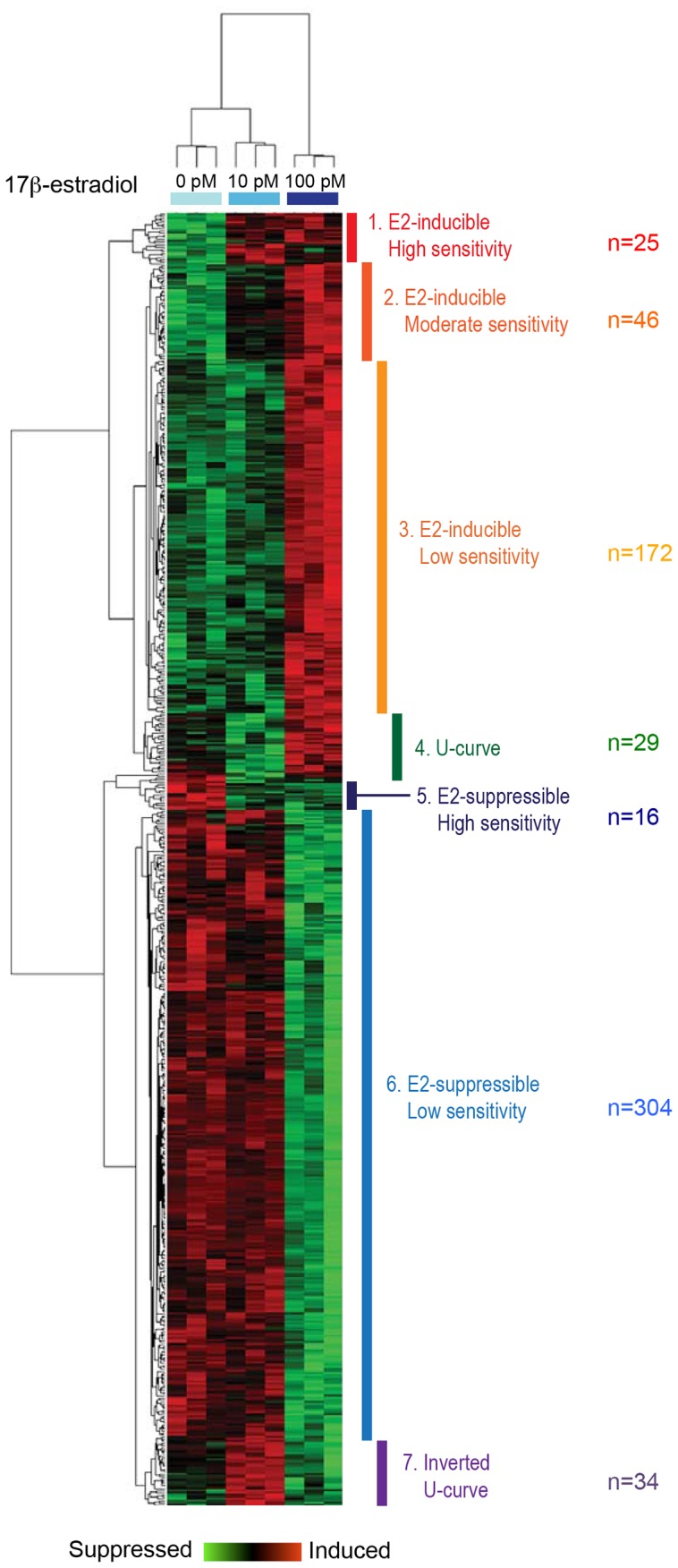
Cluster analysis of estrogen-responsive genes in fetal UGS mesenchyme cells after estrogen treatment with the two lower doses, showing strong separation of responses to control, 10 pM E2, and 100 pM E2 treatments. Based on clustering, genes were identified as falling into one of 7 groups that differed in their responses to low-dose E2.

These 628 genes were subjected to hierarchical clustering, which classified them into seven groups, based on induction or suppression of gene activity and on relative sensitivity to E2 (Figure 1). The seven groups were categorized as: 1) E2-inducible, high sensitivity genes; 2) E2-inducible, moderate sensitivity genes; 3) E2-inducible, low sensitivity genes; 4) U-shaped dose-response genes; 5) E2-suppressible, high sensitivity genes; 6) E2-suppressible, low sensitivity genes; and 7) Inverted U-shaped dose-response genes. Gene Ontology (GO) analysis was performed on these groups of genes using g:Profiler [22], [23] and DAVID [24], [25].

**Figure 2:**
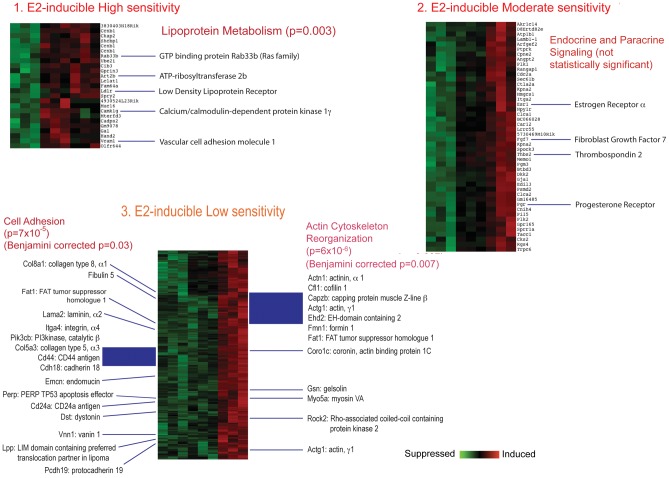
Detail of E2-inducible genes in groups identified by clustering analysis. Set 1: E2-inducible high sensitivity. Set 2: E2- inducible moderate sensitivity. Set 3: E2-inducible low sensitivity. Select genes of interest are highlighted. The figures show raw p values as well as, where indicated, Benjamini-Hochberg corrected p values.

**Figure 3:**
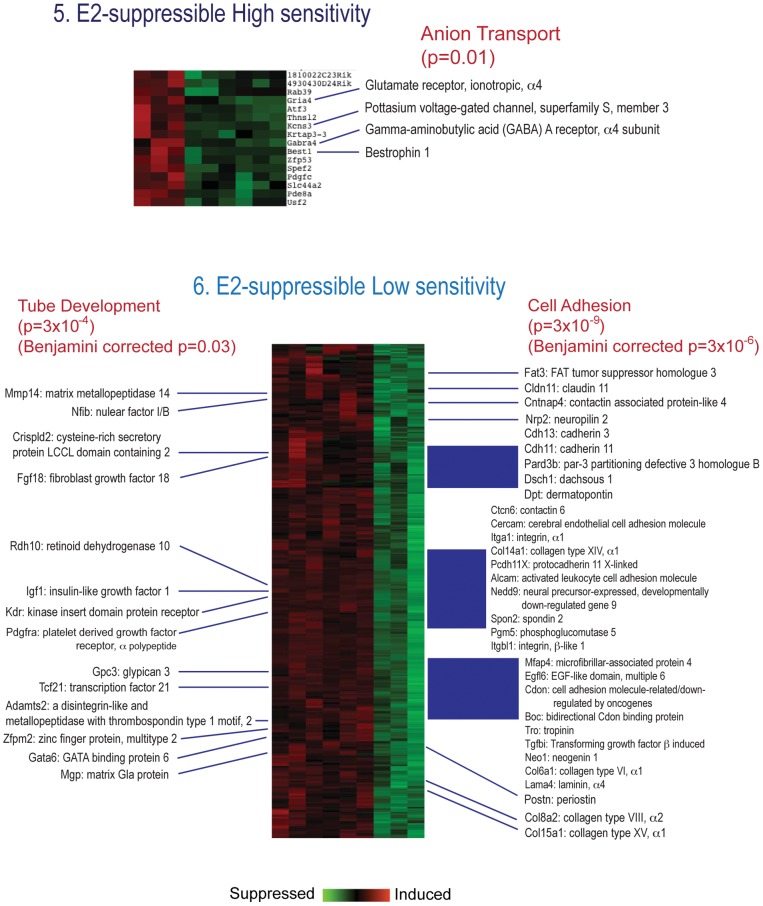
Detail of E2-suppressible genes in groups separated by clustering analysis. Set 5: E2-suppressible high sensitivity. Set 6: E2-suppressible low sensitivity. The figures show raw p values as well as, where indicated, Benjamini-Hochberg corrected p values.

Analysis of GO enrichment of E2 inducible genes (Figure 2) indicated effects on pathways for Cell Adhesion, EGF-like Calcium Binding, Sterol Biosynthesis, and Actin Filament & Cytoskeleton. Results for all E2-inducible genes, from groups 1, 2 and 3, were similar, and together suggested changes in cell adhesion, morphology, and sterol biosynthesis. Analysis of genes with a U-shaped dose response (Group 4, data not shown) did not yield specific GO/pathway effects. Analysis of E2- suppressible genes (Figure 3) indicated no specific pathway effects within the highly sensitive gene set (Group 5), but nor indicated significant effects within Group 6, the E2-suppressible, low sensitivity genes, on pathways related to extracellular matrix, Cell adhesion, EGF-like growth factor binding, IGF binding, Thyroglobulin, Thrombospondin, Ossification, and Somatomedin B. Overall, these pathways suggested changes in cell adhesion and reduced growth factor signaling.

**Figure 4:**
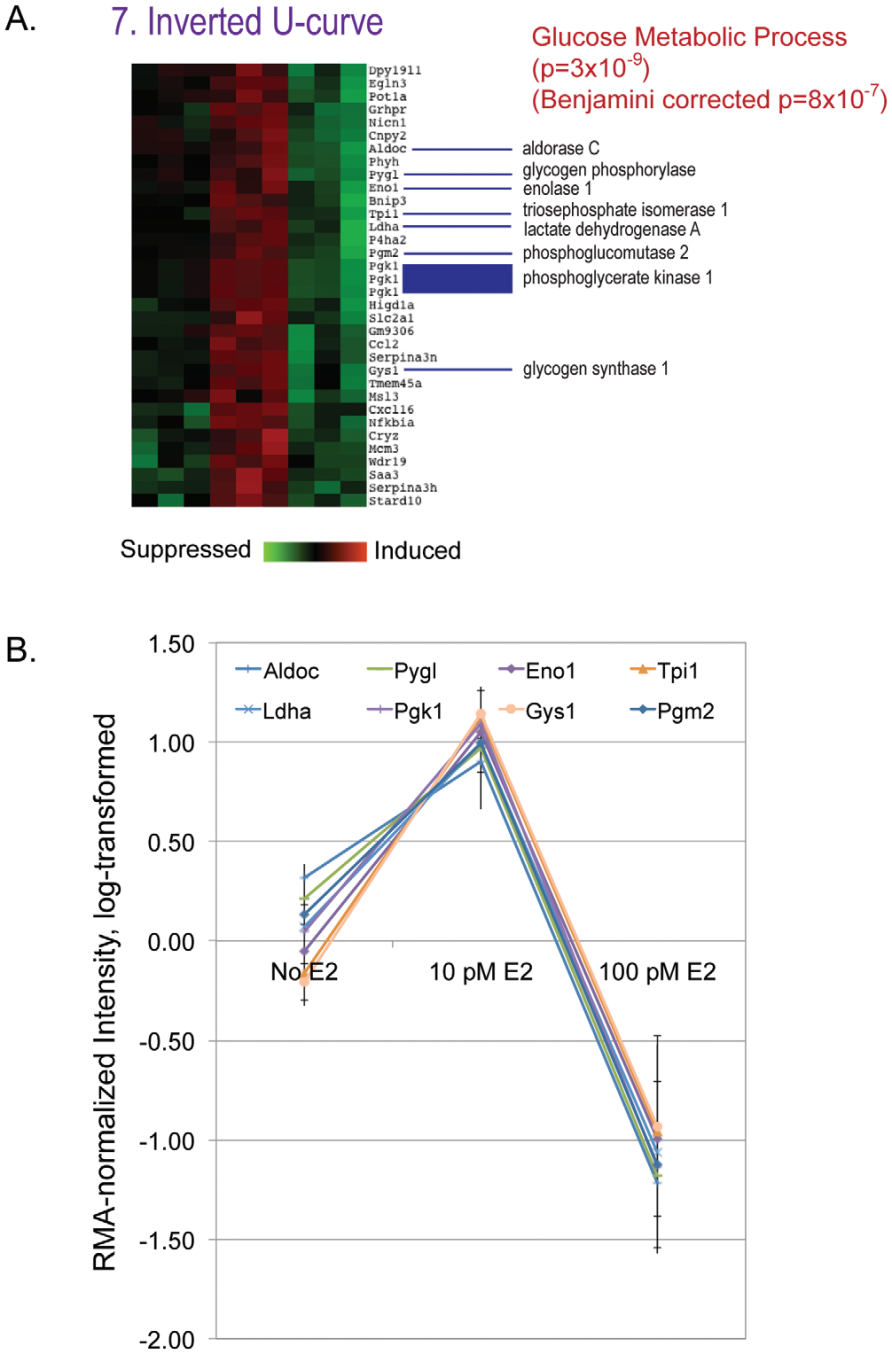
Detail of E2-suppressible genes in groups separated by clustering analysis. A) Set 7: Inverted U-curve. Both raw p values and Benjamini-Hochberg corrected p values are given. B) Genes identified as part of the glucose metabolic pathway in panel A depicted as relative values to illustrate the high association between dose and gene expression.

**Figure 5:**
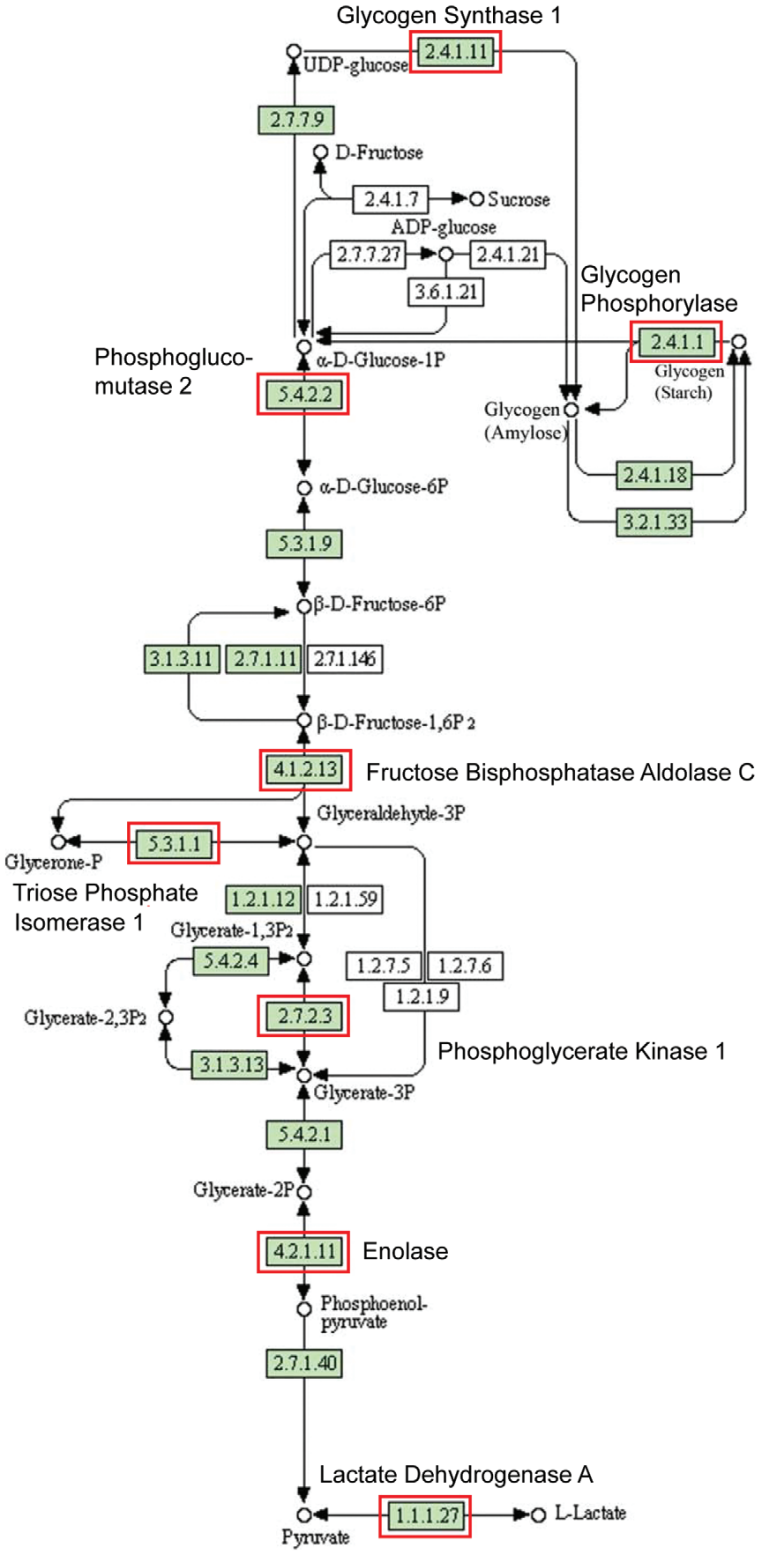
Glucose metabolism pathway. Highlighted genes (outlined in red) were influenced by lower dose estradiol treatment (10 pM and 100 pM) in an inverted U manner, suggesting enhancement of glycolysis by 10 pM E2 but suppression by 100 pM E2.

For the 34 genes showing an inverted U-shape dose response (Figure 4A), pathway analysis strongly indicated effects on sugar metabolism. Synchronized changes in mRNA expression of key genes suggest enhancement of glycolysis by 10 pM E2 but significant suppression by 100 pM E2. (Figure 4B). Specific effects within the Glycolysis pathway are illustrated in Figure 5.

### Microarray analysis of effects of a high (100 nM) concentration of estradiol on gene expression in fetal mouse prostate cells

After filtering the data in GeneSifter, using a 1.5-fold expression ratio criterion between control and estradiol treatment and a statistical cutoff at P≤0.05, and discarding genes with expression levels less than 10 fluorescence units in both treated and control samples, it was determined that 181 genes were activated by 100 nM E2 exposure and 86 genes were repressed.

The results of Gene Ontology functional enrichment analysis, within the categories of Biological Process and Molecular Function, are shown in Table 1, and categories significantly affected by the 100 nM E2 treatment were identified by z-scores. These included effects on growth, reproductive processes, and metabolic processes, and generally indicated effects of E2 on promotion of growth and inhibition of apoptosis. Table 2 lists genes in selected signaling pathways influenced by E2 treatment, again identified using z-scores. Additional genes affected by E2 treatment, selected as being “of interest” in these and other pertinent pathways, were identified manually. These data indicated significant effects of 100 nM E2 treatment on three key pathways: cell communication, androgen and estrogen metabolism, and the TGF-β signaling pathway. E2-regulated genes were also identified in other pathways of interest, namely the Wnt signaling pathway, cytokine-cytokine receptor interaction, sonic hedgehog signaling and apoptosis.

**Table 1:** Functional characterization by gene ontology (GO) terms of gene expression profiles in cells treated with 100 nM E2.

		Number of genes		z-scores	
Category	Gene Ontology Term	up-regulated	down- regulated	up-regulated	down- regulated
Biological process	metabolic process	121	69	−2.08*	−1.43
	biological regulation	100	40	2.70**	−0.99
	growth	17	3	5.11**	−0.03
	reproduction	11	9	0.94.	2.02*
	reproductive process	5	6	0.42.	2.42*
	rhythmic process	6	1	3.92**	0.23
Molecular function	catalytic activity	135	76	−2.3*	0.82
	transporter activity	53	35	2.76**	1.42

### Confirmation of estrogen-regulated genes

**Figure 6:**
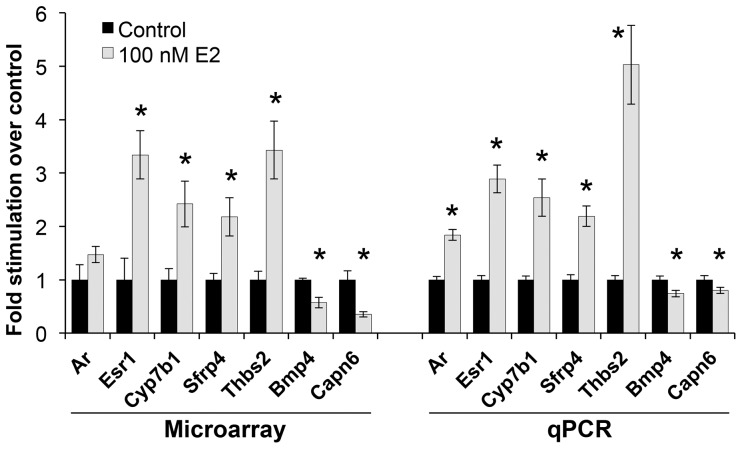
Comparison of expression of selected genes measured by microarray and by Q-PCR. Gene expression in cells treated with 100 nM 17β-estradiol (grey bars) is compared to that in untreated control cells (black bars). * Control vs. treated cells statistically different, p<0.05. The qPCR data were previously published elsewhere [19].

The expression patterns of several genes demonstrated to be up- or down-regulated by estradiol treatment using microarray analysis were validated in independent samples using quantitative PCR. The genes selected were: Ar, Bmp4, Capn6, Cyp7b1, Esr1, Sfrp4, and Thbs2. Ar and Esr1 were chosen because we have shown by qPCR [14] that estradiol stimulates Ar and Esr1 mRNA expression. The other genes were selected based on strength of response and relevance to cell growth. In this particular study, the effects of estradiol on Ar expression, while in the same direction as predicted by earlier studies, did not quite reach significance by microarray analysis. The results of the follow-up qPCR analysis are shown in Fig. 6. The data obtained for cells treated with estradiol are consistent with the microarray expression profiles.

## Discussion

The effects of fetal E2 exposure on prostate development do not follow a monotonic dose-response [11], [12]. Previous studies have shown that exposure of male mouse fetuses to a very small increase in serum E2 [11], or to very low maternal doses of the estrogenic drugs DES and ethinylestradiol or the xenoestrogen BPA, lead to basal epithelial cell hyperplasia and to a permanent increase in prostate AR binding activity, resulting in an increase in prostate size in adulthood [11], [12], [13], [26]. Those findings showed that at low doses, estrogen has a stimulatory effect on the action of androgen in regulating prostate differentiation and subsequent prostate function, including development of early stage prostate cancer in adulthood [27], [28]. In contrast, opposite effects have been found at much higher doses of E2 and xenoestrogens. Prenatal or neonatal exposure of rats or mice to high doses of estrogens led to a decrease in prostate growth during the time of exposure in development, which led to reduced prostate size and androgen responsiveness in adulthood [3], [11], [12], [13], [29], [30].

Non-monotonic dose responses were seen in our initial examination of the effects of estradiol and BPA on Ar and Esr1 expression in fetal mouse UGS mesenchyme [14], and dose-related variation in the pattern of gene expression was also observed for a large number of genes in human MCF-7 breast cancer cells, in response to doses of E2 between 10–100 pM [31]. Because of these prior findings as well as different *in vivo* effects of high and low doses of estrogen, we chose to examine the effects of E2 on gene expression in fetal prostate mesenchyme cells by microarray analysis, using two low doses (10 pM and 100 pM) as well as a high dose (100 nM) that had resulted in maximal Ar expression in our prior study with the same fetal mesenchyme cells [14]. In laboratory rats and mice, the free serum concentration of E2 (unbound to plasma proteins and unconjugated) is about 2 pM during the initial period of prostate development [11] although calculation of the actual biologically active fraction of E2 during sexual differentiation is complicated by uncertainty regarding the bioavailability of albumin-bound E2 and the capacity for the maternal-placental-fetal tissues to deconjugate sulfated estrogens [32]. Total serum E2 during this period is in the range of 300 pM [33], and thus the low doses of E2 used in this study are physiologically relevant.

These microarray experiments were performed as a hypothesis generation step for a study of effects of estrogens on prostate development and differentiation, and the sample size is small. Because of this, the data must be seen as preliminary, but the results do indicate activation of different patterns of gene expression and dominance of different pathways at low, physiologically relevant, compared to high, pharmacological, doses of E2. Results from the lowest (10 pM and 100 pM) doses of E2 treatments indicate E2-inducible genes within pathways related to cell adhesion, actin cytoskeleton reorganization, EGF-like calcium binding, sterol biosynthesis and lipoprotein metabolism, and E2-suppressible genes within pathways related to growth factor signaling, tube development and additional effects on cell adhesion. At the high (100 nM) concentration, E2 induced genes enriched for steroid hormone signaling and metabolism, cytokines and their receptors, cell-to-cell communication, and TGF-β signaling (Table 2). Results from the 100 nM E2 treatment thus indicated effects on cell adhesion pathways, but also emphasized a stimulation of a positive feedback loop involving steroid hormone receptors and genes related to growth and metabolism that promote rather than inhibit cell growth. Taken together, these results suggest that fetal prostate mesenchymal cells may regulate epithelial cells through direct cell contacts when estrogen levels in mesenchyme are in the pM range, whereas growth factors might play significant roles when estrogen levels are higher in the nM range.

**Table 2:** Effects of 100 nM estradiol treatment on gene expression within specific regulatory pathways identified as being of interest.

Pathway/Category	Direction	Ratio	Gene Identifier	Gene Name
Cell Communication	Up	2.50	AV239646	Gjb2
*z-score (up) = 3.32*	Up	1.96	BE197934	Krt1-14
	Up	2.62	AV330726	Gja1
	*Up*	*3.16*	*BC006894*	*Gja1*
	*Up*	*3.81*	*M63801*	*Gja1*
	Up	4.48	L06421	Thbs2
	*Up*	*3.43*	*NM_011581*	*Thbs2*
	Up	1.83	BI455189	Col6a2
Androgen and estrogen metabolism	Down	7.25	NM_023135	Sult1e1
*z-score (up) = 2.4*	Up	2.42	NM_007825	Cyp7b1
	Up	8.12	NM_01378	Hsd17b9
TGF-beta signaling pathway	Down	1.93	NM_010496	Idb2
*z-score (down) = 2.19*	Down	1.83	NM_008046	Fst
	Down	1.75	NM_007554	Bmp4
	Down	3.27	BM230984	Tgfb14i
	Up	3.67	BB353211	Inhbb
	Up	4.48	L06421	Thbs2
	*Up*	*3.43*	*NM_011581*	*Thbs2*
Steroid hormone receptors	Up	3.34	NM_007956	Esr1
	Up	5.29	NM_008829	Pgr
Wnt signaling	Down	2.22	NM_009519	Wnt11
	Up	3.72	NM_009526	Wnt6
	Up	1.89	W29605	Wnt7b
	Up	5.85	NM_020265	Dkk2
	Up	2.18	BB221995	Sfrp4
Cytokine-cytokine Receptor interaction	Up	2.77	NM_019583	Il17rb
	Up	2.21	NM_011330	Ccl11
	Up	6.53	NM_021443	Ccl8
	Up	3.67	BB353211	Inhbb
	Up	3.85	AF000304	Il4ra
	*Up*	*2.91*	*NM_010557*	*Il4ra*
Hedgehog signaling	Down	2.22	NM_009519	Wnt11
	Down	1.75	NM_007554	Bmp4
	Up	3.72	NM_009526	Wnt6
	Up	1.89	W29605	Wnt7b
Apoptosis	Up	2.07	BF137345	Birc4
	Down	2.85	NM_007603	Capn6
	*Down*	*2.23*	*AI747133*	*Capn6*
Prostate cancer	Up	3.40	BC010786	Creb3l3
	Up	2.21	AJ252157	Foxo1
	Up	2.60	NM_019739	Foxo1
Basal cell carcinoma	Down	2.22	NM_009519	Wnt11
	Down	1.75	NM_007554	Bmp4
	Up	3.72	NM_009526	Wnt6
	Up	1.89	W29605	Wnt7b

All genes listed are significantly altered at P<0.05. Where the z-score for the entire pathway was significant, the score is given below the pathway name. Where multiple probes for the same gene are represented in these lists (indicated by italics), agreement was good between the probes.

Importantly, an inverted U (non-monotonic) response was seen within the low-dose results, with enhancement of glycolysis observed at 10 pM E2 but significant suppression at 100 pM E2 (Figure 5). The expression of these specific genes was not influenced by 100 nM E2, indicating that the stimulation of glycolysis is highly dependent on dose and only seen at low pM E2 concentrations. This is of particular interest given the Warburg effect, the observation that most cancer cells rely on glycolysis to generate the energy needed for cellular processes, in contrast to normal differentiated cells that use mitochondrial oxidative phosphorylation [34]–[35]. The enhancement of glycolysis seen in our culture was only at the lowest dose tested here, 10 pM (2.72 pg/ml), and as such is intriguing because mice exposed prenatally to a very similar concentration of estradiol have enlarged prostates in adulthood [11] relative to mice exposed to higher doses. It is interesting to speculate on whether there is a relationship between the enhancement of cell proliferation rate and glycolysis seen in cancer cells, and the enhancement of glycolysis in fetal prostate mesenchymal cells and increased prostate size due to hyperplasia seen in mice.

Only 29 genes out of those screened were influenced by all doses of estradiol examined (Table 3). For approximately half of these genes the dose-response relationship was monotonic, although some of these were maximally up- or down-regulated at the 100 pM dose. For the rest, the direction of the effect (stimulation or suppression of gene expression) was either strongly reversed at the highest (100 nM) E2 concentration (a non-monotonic response), or simply showed a suggestion of reversal at the highest dose. Of the monotonic profiles, two genes showed particularly strong linearity with dose: Angpt2 (angiopoetin 2) and Sprr1a (small proline-rich protein 1a). Angpt2 expression is strongly correlated with prostate cancer progression [36] and is stimulated by growth factors, especially VEGF [37]–[38]; Vegf expression is stimulated by androgen treatment in fetal prostate fibroblasts [39], but we did not observe an effect of estrogen on Vegf expression here. Expression of Sprr genes is typically restricted to cells committed to terminal differentiation [40]. Although strong up-regulation of Sprr1a has been associated with abnormal cell differentiation in uterine tissue from neonatal CD-1 mice treated with diethylstilbestrol [41], effects in the developing prostate have not previously been reported.

**Table 3:** Genes whose expression was significantly (P≤0.05) influenced by estradiol (E2) treatment at all doses tested.

		Log2 fold expression relative to control				
Low-dose cluster group	Gene ID	No E2	10 pM E2	100 pM E2	100 nM E2	Monotonic trend?
Inducible_moderate	Sprr1a	0.00	0.98	2.23	5.57	Y
Inducible_moderate	Angpt2	0.00	1.20	2.25	3.77	Y
Inducible_moderate	Dkk2	0.00	0.67	2.21	2.55	Y
Inducible_moderate	Pgr	0.00	0.98	2.22	2.40	Y
Inducible_low	Fabp7	0.00	0.75	2.18	2.26	Y
Inducible_low	Fbxo32	0.00	−0.33	1.52	1.57	Y
Inducible_moderate	Esr1	0.00	1.00	2.19	1.74	I
Inducible_moderate	Rgs4	0.00	0.64	2.09	1.81	I
Inducible_moderate	Thbs2	0.00	1.49	2.20	1.78	I
Inducible_moderate	Btbd3	0.00	0.95	2.25	1.05	N
Inducible_moderate	Gja1	0.00	0.66	2.21	1.51*	N
Inducible_moderate	Npy1r	0.00	1.14	2.16	1.49	N
Inducible_low	Perp	0.00	0.21	2.06	0.71	N
Suppressible_low	Sult1e1	0.00	0.20	−1.81	−2.86	Y
Suppressible_low	Lcn2	0.00	0.49	−1.63	−2.44	Y
Suppressible_low	Egfl6	0.00	−0.20	−2.04	−1.93	Y
Suppressible_low	Pdlim3	0.00	−0.14	−1.96	−1.95	Y
Suppressible_low	Cdkn1c	0.00	−0.19	−1.99	−1.16	Y
Suppressible_low	Capn6	0.00	−0.43	−1.93	−1.51	I
Suppressible_low	Igfbp2	0.00	0.15	−1.87	−0.96	I
Suppressible_low	Wnt11	0.00	−0.55	−2.11	−1.15	I
Suppressible_low	Cyb561	0.00	0.57	−1.52	−0.93	I
Suppressible_low	Gda	0.00	−0.49	−2.06	−0.77	I
Suppressible_low	Dpep1	0.00	0.97	−1.19	−0.67	I
Suppressible_low	Zfp161	0.00	0.10	−1.70	0.74	N
Suppressible_low	Sfrp4	0.00	0.16	−1.77	1.12	N
Suppressible_low	Penk1	0.00	−0.53	−1.95	1.06	N
Suppressible_low	Enpp2	0.00	0.90	−1.17	1.85	N
U-curve	Cd80	0.00	−1.04	1.12	0.65	I

For each gene the log2 value of the fold change is given, and thus up-regulated and down-regulated genes are reflected in positive and negative numbers respectively. Genes are sorted first according to the cluster groups identified for the low-dose treatments (see Methods), and then by whether the trend at the high dose (100 nM) is consistent with the results seen at lower doses. Y = monotonic trend; gene expression at 100 nM E2 continues the trend at lower doses or has reached a plateau at that point. N = trend is clearly not monotonic; gene expression at 100 nM E2 is in the reverse direction of the trend at lower doses. I = suggestion of non-monotonic trend; gene expression at 100 nM shows slight reversal of trend at lower doses. *Value is average value for all probes for this gene (n = 5).

Also of interest in this 29-gene subset are the clear inverse U effects on Perp and Gja1 expression. Perp is typically upregulated during apoptosis [42] but is also important for promoting desmosomal cell-cell adhesion [43], and loss of Perp is associated with dysregulation of cell adhesion and promotion of tumor development and progression [44]. Decreased expression of Gja1 (Cx43) is similarly consistent with loss or reduction of cell-cell communication. Only one gene in this 29-gene subset, Enpp2, showed a U-shaped response to increasing E2 concentrations; Enpp2 codes for autoaxin, an ecto-enzyme responsible for producing lysophophatidic acid (LPA), known to be a mitogen for both ovarian and prostate cancer cells, which stimulates cell proliferation, survival and migration (reviewed in [45]). These non-monotonically expressed genes reinforce the general conclusion that pathways related to cell adhesion are influenced by estrogen treatment, but also suggest a different effect of the highest dose relative to the lower doses, with a progression toward increased cell proliferation and migration at increasing dose.

The Wnt signaling pathway was influenced at all E2 doses examined, but with an emphasis toward up-regulation of canonical Wnt/β-catenin stabilization signaling at the high dose, and non-canonical (PCP) signaling at lower doses. The high-dose effect may be mediated through the known association of β-catenin with AR and ER. Truica *et al.* have shown that β-catenin significantly enhances androgen-stimulated transcriptional activation by the AR, and that β-catenin also increases AR transcriptional activation by E2 [46]. Although many Wnt genes are differentially expressed in the prostate according to age [47], their role in prostate development, and particularly their interactive and temporal roles, is only starting to be described.

At the high dose of E2 we observed changes in genes related to steroid hormone metabolism, and alterations in steroid hormone signaling that would lead in turn to disruption of the normal expression of other developmentally important genes. Of particular interest was the observed up-regulation of Cyp7b1, which catalyzes the metabolism of the DHT metabolites 3α-Adiol and 3β-Adiol, and is thought to control cellular levels of both androgens and estrogens [48]. We verified by quantitative PCR (qPCR) that the up-regulation of Esr1 observed in these estrogen-treated cells was dose-dependent and consistent with our prior data ([14], data not shown); up-regulation of Ar was seen by qPCR but did not reach statistical significance by microarray. Esr1 was stimulated across the entire E2 dose range in this study, and thus is a potential common mechanism for the initiation of consequent signaling events. Stimulation of Esr1 and Ar serve to amplify estrogen and androgen signaling respectively, and in the intact gland there would be further potential for signal amplification, with local conversion of testosterone not only via Srd5a1 to the more potent androgen DHT, but also via aromatization to E2.

It is important to note that the intracellular concentration of E2 within the urogenital sinus during development is still unknown. The dose of E2 that reaches ER in male mouse UGS mesenchyme cells would depend not only on E2 uptake from the blood but also on local aromatization of testosterone to E2. Because of this issue, we administered E2 over a wide dose range, but also ensured that the opportunity for aromatization was controlled by the use of DHT rather than testosterone in the culture medium. Total testosterone circulates in the range of 5–8 nM in the male rat and mouse fetus during prostate differentiation [11], [32]. Because there is no high-affinity testosterone binding protein in the blood at this time, and testosterone is only weakly bound to albumin, the result is that the percentage of total testosterone in blood that is bioactive is high, particularly compared to E2, which binds to the high-affinity plasma protein alphafetoprotein. Serum testosterone thus provides a substantial pool from which intracellular E2 can be formed by aromatization in fetal prostate mesenchyme cells [32], [49]. Arase and colleagues [50] have measured E2 concentrations in fetal male mouse UGS tissue at GD17 and postnatal day (PD) 1, which approximated 10 and 25 pg/g, respectively. These concentrations are consistent with the low doses of E2 that we administered in this study, although again we do not know how much of this E2 reaches ER (the actual dose at target). Future work should address the dynamics of estrogen concentration and receptor activation both *in vitro* and *in vivo*.

The up-regulation of Pgr by all doses of E2 administered here to UGS mesenchyme cells is in general agreement with Risbridger et al., who reported up-regulation of progesterone receptors (PR) in the adult mouse prostate after estrogen treatment [51], and with data from Nishino et al. that showed enhancement of progesterone's proliferative effects on the adult rat prostate after co-treatment with E2 or DHT [52]. The presence of PR may be more relevant during fetal life, when progesterone levels are higher, than in adulthood when progesterone levels are low. The issue of fetal responsiveness to progestins is complex in that there is evidence that progestins can have anti-androgenic influences on sexual differentiation, through inhibition of 5α-reductase [53], [54]. Up-regulation of Pgr is thus a potential mechanism for disruptive effects of estrogens on male accessory reproductive organ development, but its impact will require further study.

Neonatal estrogen treatment is known to affect the expression of several genes critical to prostate development. Notable examples are Hoxb13, Nkx3.1, Shh, Fgf10 and Bmp4 [55]. Some of the genes that responded to E2 treatment in our cells agree with the findings of others (Hoxb13, Bmp4), but several of the “candidate” genes were not affected at the doses we examined. There may be several reasons for this, but two are critical. First, we deliberately cultured only the mesenchyme cells, to specifically examine effects of E2 on gene expression in the cells that initiate early prostate differentiation. Without the two-way communication that occurs between epithelial and mesenchymal cells in the developing prostate the full range of gene expression will not be seen [1], [56], [57]. For example, Nkx3.1 is expressed only in epithelial cells in regions of ductal growth, although its expression is dependent on the presence of UGS mesenchyme [58]. Similarly, Ptc and Gli, components of the Shh signaling pathway that are important for directing ductal growth, are expressed in the mesenchyme but are regulated by Shh signaling from the epithelium [59]. Additionally, in studies performed *in vivo*, other factors provided via blood circulation (known or unknown), as well as shifts in hormone levels that occur during late fetal life, parturition and early postnatal life [32], [59], will influence gene expression. Consequently, studies performed in whole tissues of intact animals are bound to yield different and more complex results.

Developmental estrogen exposure has the potential to acutely stimulate abnormal growth and induction of hyperplasia in the developing prostate [12], and this clearly establishes the potential for abnormal function in later life and a predisposition toward adult prostate disease [28]. The growth of fetal prostate epithelial cells and duct formation are driven by signals from the UGS mesenchyme [9], and our results suggest that the developmental effects of estrogens or xenoestrogens on UGS differentiation may be mediated initially by enhanced mesenchymal cell responsiveness to sex steroid hormones through up-regulation of steroid hormone receptor concentrations, with subsequent effects on other genes that differed based on the dose of E2. The differing patterns of gene expression at low and high E2 concentrations and the presence of non-monotonic responses of some genes to the wide (10,000-fold) range of E2 concentrations studied are consistent with non-monotonic dose effects on prostate development *in vivo* [11], [12], [13].

## Supporting Information

Article S1 **Previously published article** [**19**] **that included qPCR data from validation tests for microarray experiments.** The data presented (Figure 5, page 89) show effects of 100 nM estradiol on expression of seven genes, and effects of 1000 nM bisphenol A on the same seven genes. Microarray data are not included.
(PDF)
