# Supplementary material for: Dose-Related Estrogen Effects on Gene Expression in Fetal Mouse Prostate Mesenchymal Cells
Source: PLoS One. 2012 Oct 29;7(10):e48311. doi: 10.1371/journal.pone.0048311 (PMC3483223; doi:10.1371/journal.pone.0048311)
Supplement: Article S1 — Previously published article [19] that included qPCR data from validation tests for microarray experiments. The data presented (Figure 5, page 89) show effects of 100 nM estradiol on expression of seven genes, and effects of 1000 nM bisphenol A on the same seven genes. Microarray data are not included. (PDF) [file pone.0048311.s001.pdf]

Journal of Steroid Biochemistry & Molecular Biology 127 (2011) 83–95

Contents lists available at ScienceDirect

Journal of Steroid Biochemistry and Molecular Biology

journal homepage: www.elsevier.com/locate/jsbmb

Review

Estrogenic environmental chemicals and drugs: Mechanisms for effects on the developing male urogenital system

Julia A. Taylor*, Catherine A. Richter[1], Rachel L. Ruhlen[2], Frederick S. vom Saal

Division of Biological Sciences, University of Missouri, Columbia, MO 65211, USA

ARTICLE INFO

Article history:
Received 21 April 2011
Received in revised form 21 July 2011
Accepted 22 July 2011

Keywords:
Prostate development
Estrogen action
Endocrine disruption
Bisphenol A

ABSTRACT

Development and differentiation of the prostate from the fetal urogenital sinus (UGS) is dependent on androgen action via androgen receptors (AR) in the UGS mesenchyme. Estrogens are not required for prostate differentiation but do act to modulate androgen action. In mice exposure to exogenous estrogen during development results in permanent effects on adult prostate size and function, which is mediated through mesenchymal estrogen receptor (ER) alpha. For many years estrogens were thought to inhibit prostate growth because estrogenic drugs studied were administered at very high concentrations that interfered with normal prostate development. There is now extensive evidence that exposure to estrogen at very low concentrations during the early stages of prostate differentiation can stimulate fetal/neonatal prostate growth and lead to prostate disease in adulthood. Bisphenol A (BPA) is an environmental endocrine disrupting chemical that binds to both ER receptor subtypes as well as to AR. Interest in BPA has increased because of its prevalence in the environment and its detection in over 90% of people in the USA. In tissue culture of fetal mouse UGS mesenchymal cells, BPA and estradiol stimulated changes in the expression of several genes. We discuss here the potential involvement of estrogen in regulating signaling pathways affecting cellular functions relevant to steroid hormone signaling and metabolism and to inter- and intra-cellular communications that promote cell growth. The findings presented here provide additional evidence that BPA and the estrogenic drug ethinylestradiol disrupt prostate development in male mice at administered doses relevant to human exposures.

© 2011 Elsevier Ltd. All rights reserved.

Contents

1. Interaction of androgen and estrogen with androgen and estrogen receptors to regulate the differentiation of the urogenital sinus 84
2. Prostate estrogen receptors 84
3. The environmental estrogenic chemical bisphenol A (BPA) 85
   3.1. BPA action via steroid hormone receptors 85
   3.2. Environmental significance of bisphenol A 85
   3.3. BPA effects on prostate 86
4. Dose dependency of estrogen effects 87
   4.1. Low-dose stimulating and high-dose inhibiting effects of estrogen on prostate development 87
   4.2. Nonmonotonic dose–response curves 88
5. Experiment to examine effects of estradiol and BPA on gene expression in fetal mouse prostate mesenchyme in primary culture 88
   5.1. Introduction 88

Abbreviations: AR, androgen receptor; Ar, androgen receptor gene; Bmp4, bone morphogenetic protein 4; BPA, bisphenol A; Capn6, Calpain; Cyp7b1, cytochrome P450, family 7, subfamily b, polypeptide 1; DES, diethylstilbestrol; DHT, 5α-dihydrotestosterone; EGF, epidermal growth factor; ERα, estrogen receptor α; ERβ, estrogen receptor β; ERR, estrogen related receptor; Esr1, estrogen receptor; Fgf10, fibroblast growth factor 10; IGF-1, insulin-like growth factor 1; Nkx3.1, NK3 homeobox 1; Q-PCR, quantitative reverse transcriptase-polymerase chain reaction; SERM, selective estrogen receptor modulator; SFRP, secreted frizzled-related protein; Sfrp4, secreted frizzled-related protein 4; Shh, sonic hedgehog; TGF-β, transforming growth factor-β; Thbs2, Thrombospondin 2; UGS, urogenital sinus; Wnt, Wingless-related MMTV integration site family; Wnt 7b, Wingless-related MMTV integration site family member 7b; Wnt11, Wingless-related MMTV integration site family, member 11.

* Corresponding author. Tel.: +1 573 882 2482/0983; fax: +1 573 884 5020.
E-mail addresses: taylorja@missouri.edu (J.A. Taylor), crichter@usgs.gov (C.A. Richter), rruhlen@atsu.edu (R.L. Ruhlen), vomsaalf@missouri.edu (F.S. vom Saal).
[1] Present address: USGS, Columbia Environmental Research Center, Columbia, MO 65201, USA.
[2] Present address: A.T.Still University, Kirksville, MO 63501, USA.

0960-0760/$ – see front matter © 2011 Elsevier Ltd. All rights reserved.
doi:10.1016/j.jsbmb.2011.07.005

5.2. Materials and methods ... 88
5.3. Results and discussion ... 89
6. Controversies concerning the stimulating effect of estradiol, bisphenol A and estrogenic drugs on the prostate in mice ... 90
7. Experiment to examine effects of ethinylestradiol administration to pregnant CD-1 mice: significant enlargement of the prostate in adult male offspring at subclinical doses ... 91
7.1. Introduction ... 91
7.2. Materials and methods ... 91
7.3. Results and discussion ... 91
8. Conclusions ... 92
Disclosure statement ... 92
Acknowledgement ... 92
References ... 92

1. Interaction of androgen and estrogen with androgen and estrogen receptors to regulate the differentiation of the urogenital sinus

The prostate, together with the urethra and associated periurethral glands, differentiates from the male urogenital sinus (UGS) in response to the binding of the 5α-reduced metabolite of testosterone, 5α-dihydrotestosterone (DHT), to androgen receptors (AR) in the UGS mesenchyme (Fig. 1). Signals from the UGS mesenchyme initiate the process of proliferation and budding of the UGS epithelium to form the initial ductal components of the prostate, and continued differentiation depends not only on androgen action but also on signaling interactions between the epithelial and mesenchyme cell populations [1,2]. The growth factors that mediate the effects of androgen on prostate development (andromedins) have been extensively studied, although there remain gaps in our knowledge [2–4]. In addition, there is now considerable evidence that estrogen, while not essential, modulates the activity of androgen in regulating prostate development and subsequent adult function and disease. Estrogens have long been implicated in prostate disease, both benign and malignant [5–7]. Estrogens cause squamous metaplasia of the prostate epithelium when unopposed by androgens [8,9]. Endogenous and exogenous estrogens influence transcriptional regulation of numerous genes that have estrogen response elements (EREs) within their promoters [10,11], including members of the insulin-like growth factor, fibroblast growth factor, hepatocyte growth factor, nerve growth factor, and transforming growth factor-β families. The signaling systems involved in mediating these effects are not fully understood [12].

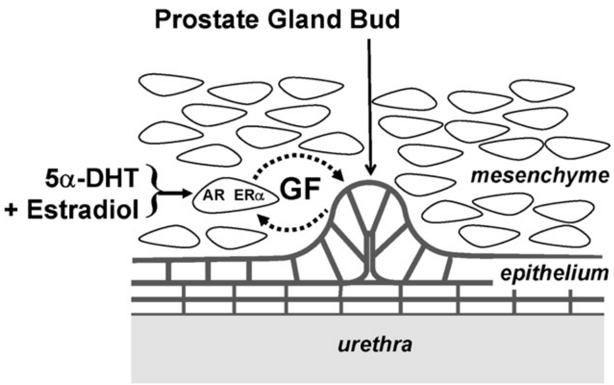

Fig. 1. Prostate differentiation is initiated shortly after the onset of testosterone secretion by the fetal testis, and is dependent on testosterone conversion to 5α-DHT. 5α-DHT binds to androgen receptors in the UGS mesenchyme and stimulates the production of growth factors and signaling molecules (collectively identified here as "GF"). Epithelial cells respond to these signals with the production of other signaling molecules, and the ensuing cross-talk between the two cell types promotes the outgrowth of epithelial cell buds into the surrounding mesenchymal cell layer and thus initiates the process of duct formation. Modulating actions of estrogen on gene expression and duct formation are via ERα receptors in the UGS mesenchyme cells.

There is much that is still not known about the mechanisms by which endogenous and exogenous estrogenic chemicals, acting via binding to classical estrogen receptors or potentially also via alternative non-classical receptor pathways, directly alter the prostate or indirectly alter the response of the prostate to androgen and other signals. Importantly, it is well known that estrogens alone do not direct prostate differentiation and growth [13]. However, prenatal or neonatal exposure of male mice to very low doses of estradiol and other estrogenic chemicals (DES, ethinylestradiol, methoxychlor, bisphenol A) stimulate prostate gland genesis and glandular growth leading to an increase in sensitivity to androgen in adulthood associated with an increase in the number of prostatic AR [14–18].

2. Prostate estrogen receptors

The discovery of estrogen receptor-beta (ERβ), cloned from a rat prostate cDNA library in 1996 [19] prompted numerous studies to determine which of the two ER subtypes mediated estrogen effects on differentiation and subsequent functioning of the prostate and other tissues. There is now considerable evidence that the modulating effects of estrogen on prostate growth and differentiation in rats and mice are mediated by ERα, while ERβ has anti-proliferative effects [11,20]. The relative roles of ERα and ERβ in human prostate gland differentiation as opposed to adult function and disease are less clear [11,21].

Prostate morphogenesis in males requires expression of AR in the UGS mesenchyme [1]. During the prenatal and neonatal period of differentiation in rodents, AR and ERα are expressed in the UGS mesenchyme [22,23] but not the UGS epithelium [20,24,25]. Recent work by Omoto et al. [20] has described precise changes in the temporal and spatial distribution of the two ER subtypes in the ventral prostate during early postnatal development. Of particular interest was the transition observed from dominance by ERα in the early postnatal period (associated with a high proliferation index) to dominance by ERβ four weeks later (associated with proliferative quiescence). Utilizing ERα and ERβ knockout mice, these authors showed that not only is early estrogen signaling required for normal prostate morphology in mice, but also the early modulating effects of estrogen on prostate differentiation are mediated by ERα, not ERβ. However, ERβ mRNA levels do increase following prostate morphogenesis, and in adult mice ERβ is highly expressed in prostatic epithelium [20], while ERα is predominantly expressed in stroma but also at low levels in the adult prostatic epithelium.

There are multiple isoforms of ERβ [26], and the tissue distribution differs between rodents and humans and changes throughout prostatic development [21]. The different roles of ERα and ERβ have been identified based on a variety of approaches in studies with rodents, but the interaction between genes regulated by these

different estrogen receptors in humans is still poorly understood. However, in the adult human prostate, evidence suggests that ligands that activate ERβ may reduce proliferation, similar to findings in mice and rats [11,20,24,27]. Our focus here will be on responses to endogenous estradiol and to xenoestrogens (drugs and environmental chemicals) that are mediated by ERα during differentiation of the UGS in males.

3. The environmental estrogenic chemical bisphenol A (BPA)

3.1. BPA action via steroid hormone receptors

Bisphenol A (BPA) is an environmental endocrine disrupting estrogenic chemical that, similar to other xenoestrogens [28], is a selective estrogen receptor modulator (SERM). It is important to note that while BPA does interact with receptors for other hormones, many of the effects that have been studied are assumed to be associated with an estrogenic mode of action [29,30]. Responses to BPA differ depending on whether ERβ or ERα is present, and also depend on the specific co-regulatory proteins associated with the receptors [31,32]. Although BPA binds ERβ with greater affinity than ERα [33], in the study by Routledge et al. the binding affinity for the receptor was not predictive of the magnitude of the response, and indicates that the examination of xenoestrogen action by in vitro binding assays alone can lead to underestimates of chemical potency. In addition, the specific tissue examined in whole cell assays used for regulatory purposes is of great importance, since one tissue (for example, the uterus) may require a very high chemical dose to show a response, as is the case with BPA, while other tissues, such as the fetal prostate mesenchyme, are far more sensitive.

The model of hormone action predicts that interactions of estrogen receptor with its co-factors are influenced by the ligand bound to ER. When ER is bound to agonists such as estradiol it interacts with co-activator proteins, while inhibition of ER occurs due to recruitment of co-repressor proteins. Effects of chemicals such as BPA are consistent with this model and lead to different responses in different tissues. The interaction of BPA with classical ERs (ERα and ERβ) also appears to result in very complex and as yet, unpredictable, outcomes in terms of whether additive or inhibitory interaction with endogenous estrogenic activity is observed. For example, in the hippocampus, BPA has the paradoxical effect of acting to block the stimulatory effects of estradiol on neuronal synapse formation [34–36]. In addition, BPA can interact with cell membrane-associated ERs that activate rapid-signaling enzyme cascades that greatly amplify responses, leading to effects at BPA concentrations at and below 1 pM [37,38].

As noted, there are estrogen receptors that activate rapid signaling systems and can alter cell function via a variety of mechanisms, including G protein-coupled receptor (GPR30 or GPER), and other non-classical membrane-associated receptors [39]. Since activation of responses via these membrane-associated ERs occurs at sub-picomolar concentrations [38,40], they have been predicted to play a significant role in mediating at least some of the "low dose" effects of BPA observed in animals [41]. However, the relative contribution of membrane-associated, cytosolic and nuclear receptors to the myriad of adverse effects associated with exposure to BPA in experimental animals, and well as the emerging findings from epidemiological studies, remains to be determined.

The orphan estrogen-related receptors (ERRs) are expressed in a variety of tissues, for example, the prostate, brain, muscle, brown and white adipocytes, heart, kidney, pancreas, placenta and breast. ERRγ is expressed in adult human prostate epithelium and has been implicated in suppression of proliferation. Low levels of expression are predictive of a poor prognosis in men with prostate cancer [42,43]. The roles of the different ERR isoforms (ERRα, ERRβ and ERRγ) in prostate development remain to be determined. In contrast to estradiol, the environmental estrogen bisphenol A (BPA), binds to ERRγ and suppresses its activity [44,45] with relatively high affinity (KD of 5.50 nM), considering that median serum levels of unconjugated (bioactive) BPA in different biomonitoring studies range from 1 to 10 nM [46] and that the bioactive concentration range is typically 10–100-fold lower than the Kd [47]. Thus, some of the endocrine-disrupting effects of BPA may occur through inhibitin of functions mediated by ERRγ, potentially reducing survival in men with prostate cancer.

BPA is an AR antagonist [48,49], with a lower affinity for AR than for either ERα or ERβ. The binding of BPA to AR is not particularly surprising, because at supra-physiological doses, other ligands for estrogen receptors, including estradiol, bind to AR, but with a relatively low affinity [50]. There is also evidence that BPA binds at low affinity to receptors for thyroid hormone, and acts to inhibit thyroid hormone action by co-repressor recruitment [51,52].

Whereas BPA has a relatively low affinity for wild-type AR, in human prostate cancer cells that express a mutant form of the AR (the AR-T877A mutant), BPA acts as a ligand for the AR mutant and stimulates cell proliferation at and below 1 nM. In addition, BPA significantly down-regulates ERβ transcriptional activity in this cell line [53]. This was interpreted as providing further evidence that ERβ antagonizes proliferation of prostatic epithelium, while ERα plays a stimulatory role in proliferation and prostate disease. Leung et al. have shown that BPA and other xenoestrogens are more similar to estradiol in terms of potency in promoting ERβ heterodimerization, important for ERβ signaling [26] than are the phytoestrogens, in spite of the fact that the phytoestrogens typically have higher affinities for both ER receptor subtypes [54].

3.2. Environmental significance of bisphenol A

Bisphenol A is a small lipophilic chemical that was synthesized in the late 19th century and was reported in 1936 to have full estrogenic activity in a rat vaginal cornification assay [55]. The discovery that BPA could be polymerized to create resins and polycarbonate plastic occurred in the 1950s, and BPA was approved for use in food and beverage containers by the FDA in 1963. Along with about 60,000 other chemicals in commerce at the time that the Toxic Substance Control Act was passed in 1976, BPA was "grandfathered" under this law as "safe" [56]. BPA is now used to manufacture a wide variety of consumer products with an estimated 8 billion pounds produced in 2008 [57].

Because of its widespread use, over the last decade BPA has become the most studied chemical with estrogenic activity. Bisphenol A leaches from products that contain it (Fig. 2), with the rate of leaching increasing as a function of age and use [58]. The rate of leaching from these products results in human exposure to BPA within the range of the low doses of BPA that we and others have used in animal experiments [46,59,60]. Based on the 2003–2004 NHANES survey, BPA is estimated to be present in urine of over 90% of people in the USA, with exposure being higher in children than in adults [61]. In vitro, experimental animal and epidemiological studies provide extensive evidence that at the levels detected in people in biomonitoring studies, which are typically in the range of 0.3–4 ng/ml [46,62], BPA can disrupt cell function, interfere with developmental processes as well as adult function, and is associated with adverse outcomes in people [63–68]. It is likely that some of these effects are mediated by response mechanisms other than classical estrogen response mechanisms [39,69]. However, as noted above, there is extensive evidence showing that many effects are consistent with a classical estrogenic mode of action [30,47]. Of particular concern is exposure of fetuses, infants and children to

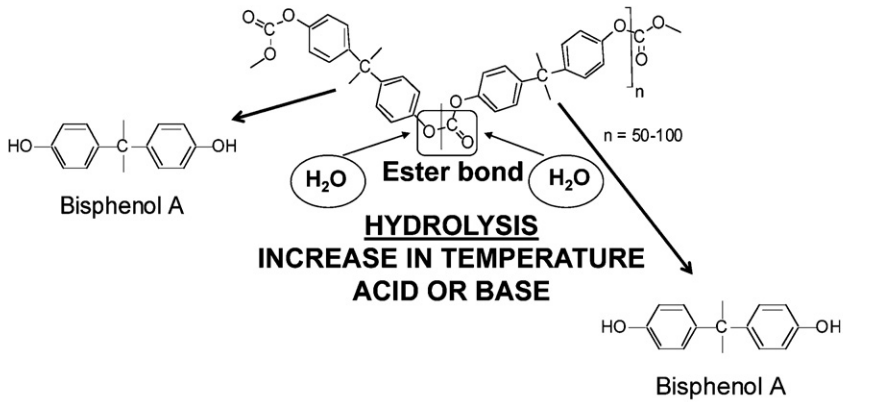

Fig. 2. Polycarbonate is composed of molecules of bisphenol A linked by ester bonds. The ester bonds are susceptible to hydrolysis when exposed to extremes of temperature or pH, and when the bonds are hydrolyzed BPA is released into the surrounding medium.

BPA, since developmental exposure can result in adverse effects that might take years to be recognized, similar to the consequence for offspring of administration of the drug diethylstilbestrol (DES) to pregnant women [70,71].

A recent analysis of data from US National Health and Nutrition Examination Survey (NHANES) 2003–2004 found that urine total BPA levels did not show the expected marked decrease as a function of fasting time [72]. This finding provides evidence that there is chronic exposure to BPA via oral and non-oral routes rather than intermittent exposure to BPA only associated with eating food or drinking beverages from BPA-containing products. In addition, there are over a dozen published studies (using a range of analytical methods) reporting levels of unconjugated BPA in adult human serum [60] that could only be achieved with much higher and chronic exposures to BPA than the estimated exposure based only on ingestion via food and drink. Our recent findings using rhesus monkeys as a model showed that a single oral administration of 400 μg/kg/day BPA was required to achieve the same levels of unconjugated BPA in adult rhesus monkeys as are found in human serum [59].

The potency of BPA relative to DES appears to be higher *in vivo* than would be expected based on comparisons of potency in MCF-7 breast cancer cells. We, and others [15,47], have shown that in MCF-7 cell culture, BPA is approximately 15,000-fold less potent than DES or estradiol. While the *in vivo* potency of BPA relative to DES is higher than expected based on the relative ability of these chemicals to stimulate proliferation of MCF-7 breast cancer cells [15], the concentration of BPA required to simulate proliferation in other human cells, such as prostate cancer (LNCaP) cells, is exactly in the concentration range of unconjugated BPA in serum of most people that have been examined (∼1–10 nM or 0.23–2.3 ng/ml) [30,46].

3.3. BPA effects on prostate

Data from a number of studies consistently show that BPA is within the range of 100–500-fold less potent than DES in the prostate [14,15,73,74]. For example, in the fetal CD-1 mouse prostate in primary organ culture, Gupta showed that a 50 pg/ml (220 pM) dose of BPA resulted in prostate enlargement, an increase in prostate gland branching, and an increase in androgen binding that was similar to the effect of a 500-fold lower dose of DES (0.1 pg/ml) [14]. We found that exposure of CF-1 mouse fetuses (via feeding the dam) to a 2-μg/kg/day dose of BPA produced a virtually identical increase in adult prostate size (Fig. 3) as a 10-fold lower dose of DES (0.2 μg/kg/day) [15,16] and a 20-μg/kg/day dose of the estrogenic insecticide methoxychlor [18]. The methoxychlor finding is interesting in that the total weight of the adult prostate was greater than that stimulated by any other estrogen in CF-1 mice. In addition, while BPA, DES and ethinylestradiol all show very similar effects on the prostate, with epithelial hyperplasia being observed in the dorsolateral prostate but not the ventral primary prostatic ducts [74], the greatest effect of methoxychlor was on the size of the ventral lobe of the prostate, which was dramatically larger than in controls.

The findings by Gupta, based on examining the fetal UGS in organ culture, demonstrate direct effects of BPA and DES on the developing UGS independent of any other potential systemic effects that these chemicals might have on maternal, placental or fetal endocrine function. These findings also show that in the fetal UGS, BPA is a more potent estrogen (relative to DES) than predicted based on the response observed in MCF-7 breast cancer cells [30] or in female rat or mouse vagina or uterus [75,76]. One reason that BPA is more potent *in vivo* relative to predictions based on in vitro assays is that BPA shows limited binding to the high affinity plasma binding protein in humans (sex hormone binding globulin or SHBG) and rodents (alphafetoprotein or AFP) [15,77,78], which is similar to prior findings for DES [79] (Fig. 4). The presence of plasma binding proteins such as SHBG significantly reduces the uptake of endogenous estrogens from plasma into cells, while chemicals such as BPA and DES bypass this barrier and thus show an increase in potency *in vivo* relative to predictions based on serum-free assay systems.

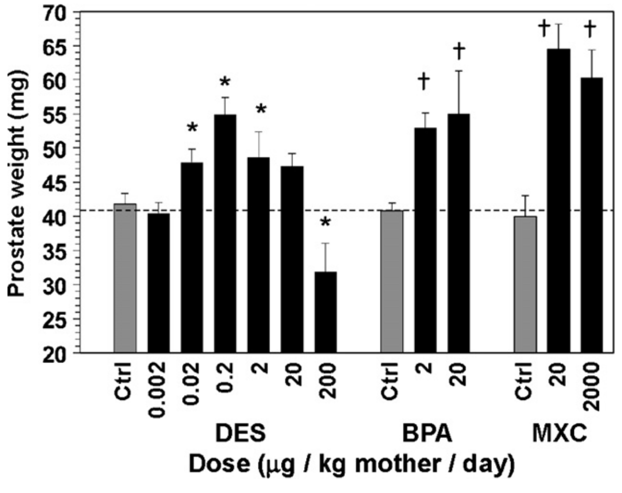

Fig. 3. Mean (+ SEM) prostate weight in 8 month-old CF-1 mice produced by females fed varying doses of diethylstilbestrol (DES), bisphenol A (BPA) or methoxychlor (MXC) from day 11 to17 of pregnancy. The dashed line represents the average prostate weight in control animals. *$p < 0.05$, †$p < 0.01$.

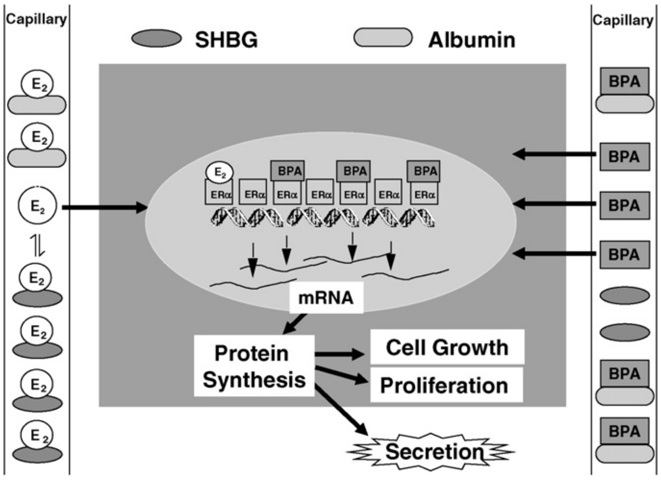

Fig. 4. Model of BPA action in blood. In humans, 17β-estradiol is primarily associated with the serum binding proteins sex hormone binding globulin (SHBG, ovals) and albumin (oblongs), and only a small fraction is unbound or free. Xenoestrogens such as BPA that do not bind well to serum proteins (BPA binds weakly to serum albumin but does not bind to SHBG at concentrations found in human serum) will have a higher free concentration in serum, and thus a higher proportion of molecules are available to reach and bind to estrogen receptors and promote estrogen receptor-mediated effects.

4. Dose dependency of estrogen effects

4.1. Low-dose stimulating and high-dose inhibiting effects of estrogen on prostate development

Until the late 1990s, most studies to examine the effects of estrogen on development of the prostate or other male reproductive organs had used very high doses of estrogenic drugs, such as DES or ethinylestradiol. For example, the effects of estrogen on development of male rat reproductive organs was studied by injecting pregnant rats with 500 μg/kg/day of DES [80], while neonatal rats were injected with approximately 1000 μg/kg/day of DES or ethinylestradiol [81]. Effects of high doses of estrogenic drugs have also been examined in mice; for example, neonatal mice were injected with 2 μg DES per pup, an approximate 500 μg/kg/day dose [82]. Results from studies of exposure to very high doses of estrogenic drugs during perinatal life led to the conclusion that estrogens inhibited prostate development, with the consequence in adulthood being a prostate about 10% of normal size, a dramatic down-regulation of androgen receptors, and epithelial dysplasia and neoplasia [82,83]. However, a clinically relevant dose of ethinylestradiol in combined oral contraceptives (fetuses are exposed to this drug when women on oral contraceptives become pregnant) is about 0.3 μg/kg/day based on a dose of 20 μg ethinylestradiol in "low dose" pills and an average body weight of 70 kg for adult women in the USA [84]. Clearly, a dose of 1000 μg ethinylestradiol/kg/day has no clinical relevance, and these high doses of estrogenic drugs (such as DES and ethinylestradiol) were also deemed irrelevant for understanding chronic low-dose exposure to environmental endocrine disruptors [85].

In contrast to this approach, we proposed that endogenous estradiol, in the low pM (pg/ml) range for total serum estradiol, altered the response of the developing prostate to androgen. This prediction was based on our finding that male CF-1 mice that were positioned *in utero* between female fetuses (2F males), which leads to elevated endogenous serum estradiol, had enlarged prostates and elevated numbers of prostatic androgen receptors as adults, relative to males that were positioned *in utero* between male fetuses (2 M males) [86]. An increase in either receptors or ligand will increase the response of an androgen or estrogen-target organ to the stimulating effect of the endogenous hormone or exogenous hormone-mimicking chemical or drug [47]. Subsequently, addition of estradiol (10 pM) was reported to increase androgen-receptor-mediated transcriptional activity induced by DHT; this was demonstrated in vitro using urogenital sinus cells co-transfected with ER and AR expression vectors [87]. In addition, estrogen and androgen have been shown to have a cooperative interaction in stimulating DNA synthesis in stromal cells obtained from hyperplastic human prostates [88], and estradiol can stimulate androgen receptor transcriptional activity in the presence of the co-activator ARA70 [50].

In the LNCaP prostate cancer cell line, which contains a mutant version of the androgen receptor, either estrogen or androgen can activate formation of a complex of AR, ER and Src, and thus induce cell proliferation through the Src-Ras-Erks pathway [89]. AR activates PAK6 kinase activity, and PAK6 inhibits transcriptional activation by AR and ER [90]. In addition, estrogen alters AR expression levels in a tissue-specific manner [91–93]. Analysis of gene expression patterns in adult human prostate stroma cells in response to a high dose of estradiol revealed hundreds of estrogen-regulated genes [94]. Estrogen treatment thus has pleiotropic effects, both *in vivo* and in vitro.

After our initial finding that a fetal mouse's intrauterine position relative to male or female fetuses was related to prostate size and number of prostatic androgen receptors in adulthood [86], we subsequently demonstrated using 1MF male CF-1 mice (males that developed between a male and a female fetus with serum estradiol levels that are intermediate between 2F and 2M male fetuses [95]) that a small experimental increase in estradiol, similar to that caused by development between female fetuses, led to an increase in prostate size and prostatic androgen receptors in adulthood [16]. In this study we examined untreated male fetuses and male offspring from females implanted with a capsule containing estradiol and used computer-assisted 3-dimensional reconstruction of the fetal prostate to show that on Day 18 of gestation in CF-1 mice, there was a significant increase in the development of prostatic ducts both in terms of the number of prostatic epithelial glandular buds and the total volume of these primary prostatic ducts as a result of exposure to supplemental estradiol. We used a highly sensitive radioimmunoassay to measure serum estradiol and a dialysis procedure we developed [96] to determine the percent of the total extractable estradiol in serum that was not bound to plasma binding proteins, which in fetal mice was found to be very low (0.2%). The concentration of total serum estradiol in control 1MF male fetuses was 94 pg/ml, while the estradiol-containing capsule led to a 52-pg/ml increase in total serum estradiol to 146 pg/ml. This 52 pg/ml increase in total serum estradiol corresponded with a 0.11 pg/ml (0.4 pM) increase in the free serum estradiol concentration in 1MF male fetuses, resulting in a free serum estradiol concentration of 0.32 pg/ml (1.17 pM) in the estrogen-treated male fetuses. As indicated above, this treatment also led in adulthood to enlargement of the prostate and an increase in the number of prostatic androgen receptors [16]. Subsequently, it was shown that neonatal treatment with a low dose of estradiol (as well as a low, 10-μg/kg/day dose of BPA) in conjunction with chronic adult treatment with estradiol and testosterone, a model initially developed with Noble rats to induce prostate cancer, led to development of high-grade prostatic intraepithelial neoplasia (HG-PIN) and differential imprinting of genes, in Sprague-Dawley rats [97,98]. These findings show that perinatal exposure to endogenous or exogenous estrogens stimulates fetal/neonatal prostate growth, with permanent consequences for prostate function and disease later in life.

Similar to effects in rodents, in adult dogs estradiol synergizes with dihydrotestosterone to increase androgen binding in prostate cells and thus increases prostate growth [99]. Studies have also shown that estradiol influences hypothalamic androgen recep-

tors in adult male rats [100]. In addition, estradiol regulates the expression of receptors for a number of hormones, such as uterine oxytocin receptors and both uterine and brain progesterone receptors [101,102]. Taken together, these findings show that the physiological effects of exposure to estrogen can include changes in the functioning of a variety of tissues due to changes in the receptors for other hormones that regulate these tissues, and this can occur throughout life. Importantly, when exposure to estrogen occurs during critical periods in development, effects on tissue function are permanent.

Interestingly, elevation of testosterone levels during development appears to have effects similar to those caused by elevation of estrogen levels. Aromatase knockout mice are unable to produce estrogen, and males exhibit increased testosterone and DHT levels in serum and tissues. These males also have enlarged prostates [103]. Thus, both an increase in serum androgen levels caused by deficient aromatase activity, and an increase in prostatic androgen receptor levels induced by elevated estrogen exposure, can lead to stimulation of prostate growth.

4.2. Nonmonotonic dose–response curves

The central assumption in toxicology has been that “the dose makes the poison”, an idea developed in the 16th century that has persisted in some communities in spite of a massive literature in endocrinology showing that this assumption is false for hormones and hormonally active drugs and chemicals [47,104,105].

There are a number of reasons why the response of tissues to estrogen and other hormones is never linear throughout a wide range of doses, and, instead, inverted-U functions are observed. First, only a very small proportion (<1%) of the total pool of estrogen receptors needs to be occupied by estradiol to stimulate a response, and proportionality of dose and receptor occupancy only occurs within a relatively narrow dose range. Second, when receptors become saturated at higher doses, an additional increase in the concentration of estrogen cannot lead to a further increase in the receptor-mediated response. In fact, exactly the opposite can occur, that is, the magnitude of response can begin to decrease. This can result, at least in part, from a process known as “receptor down-regulation” in which prolonged exposure to a high concentration of a hormone can result in the loss of active receptors [106]. Another reason for non-linear responses to estradiol (and other estrogenic chemicals) is that, at higher than physiological concentrations, they can also bind to receptors for other steroids (typically antagonistic to the natural ligand for the receptor). For example, as noted above, at supra-physiological concentrations, estradiol binds to receptors for testosterone [50,107]. High-dose studies with endocrine disrupting chemicals can thus potentially lead to “cross-talk” with receptors for other hormones, which will not be occupied at biologically meaningful levels at low doses. Finally, as dose increases, the array of genes activated and inhibited is markedly different, demonstrating that effects at high doses are distinct from effects at lower doses at the transcriptional level [108,109].

In the experiment in which we administered a low dose of estradiol to pregnant female CF-1 mice via Silastic capsule, and found that both the fetal and adult prostate was enlarged [16], we also examined a wide dose range for estradiol (via Silastic capsule) and DES (via oral administration) administered during the last half of pregnancy (during fetal sexual differentiation). For both estradiol and DES, the response to the low doses was stimulation of prostate growth, while inhibition of normal prostate development occurred at the highest doses. The inverted-U dose–response relationship for fetal DES and adult prostate weight is shown in Fig. 3 [16]. We replicated this experiment with CD-1 mice administered low and high doses of DES using computer-assisted reconstruction which demonstrated that these effects could be detected in the fetal prostate. In this experiment we showed that the stimulatory effects of low doses of estrogenic chemicals (in addition to DES, ethinylestradiol as well as BPA), were primarily on proliferation of basal epithelial cells in the primary prostatic ducts in the dorsolateral prostate, but not the ventral prostate. In addition, feeding pregnant female mice a high (200 μg/kg/day) dose of DES from gestation days 14–18 completely inhibited development of the primary prostatic ducts when fetuses were examined on gestation days 19, the day of parturition [74].

5. Experiment to examine effects of estradiol and BPA on gene expression in fetal mouse prostate mesenchyme in primary culture

5.1. Introduction

We previously reported that the endogenous estrogen 17β-estradiol as well as BPA stimulated up-regulation of both Ar and Esr1 gene expression in fetal mouse prostate mesenchyme cells in primary culture, and that these effects were antagonized by antiestrogens [110]. In the present study we sought to extend these data to examine other estrogen-regulated candidate genes in fetal mouse prostate mesenchyme using the dose of estradiol that resulted in maximum stimulation of Ar expression based on our prior study [110].

One of the problems with determining the dose of estradiol to use in cell culture is the lack of information concerning the estradiol dose at the receptor that will occur *in vivo* in relation to the dose detected in serum. Thus, while we have measured the fraction of estradiol in serum that is unconjugated and unbound to either low-affinity (albumin) or high affinity (alphafetoprotein) plasma binding proteins in fetal mice and rats [16,96], this does not provide information concerning the concentration of estradiol that can reach the estrogen receptor in the tissues that contain the enzyme aromatase (CYP19A1) in fetuses whose mothers are treated with estradiol or BPA. Male mouse and rat fetuses circulate testosterone and androstenedione in the low ng/ml range, and these androgens serve as the substrates for aromatase and estrogen synthesis (estradiol and estrone, respectively) in cells [111].

The complexity of this issue was revealed by findings reported in a study in which pregnant mice were fed 20-μg/kg/day BPA from GD 13–16. This low dose of BPA was found to stimulate a doubling of aromatase activity in UGS mesenchyme (examined on the day of birth) associated with a doubling of estradiol per gram of tissue [112]. While it has been known for some time that the developing prostate contained aromatase [111], this is the first report that an environmental estrogenic chemical could significantly stimulate aromatase activity and lead to greater tissue concentrations of estradiol relative to what would normally enter the tissue from the circulation. We previously reported that administering pregnant mice a low dose of DES (0.1 μg/kg/day) significantly increased serum estradiol levels in fetuses but not the mother [113]. Taken together, these findings suggest that there is an unexpected positive feedback effect of estrogen on aromatase activity in fetal tissues that can lead to elevated estradiol in tissues [112] that can even be detected in the systemic circulation [113].

5.2. Materials and methods

Since we lack information about the actual concentration of estradiol that is available to reach the estrogen receptor in mesenchyme during differentiation of the fetal prostate, in the present study we chose to examine by qPCR the dose of estradiol (as well as BPA) that gave the maximum increase in Ar and Esr1 gene activity in fetal mouse prostate mesenchyme in primary culture in our prior

study [110]. The concentration of estradiol chosen was 100 nM, and that of BPA was 10-fold higher (1000 nM), since BPA is less potent than estradiol.

Detailed methods for the preparation of cells and of the cell culture were previously described [110]. Briefly, the prostatic region of the UGS of gestation day 17 male fetuses was removed, the tissue was disrupted by collagenase treatment, and the dissociated mesenchymal cells were cultured. Cells were treated in vitro for four days with either 17β-estradiol (100 nM), BPA (1000 nM) or the ethanol vehicle (0.05%) alone, for four days with daily medium changes. DHT was maintained at 690 pM (200 pg/ml) throughout. At the end of the treatment period the cells were washed once with PBS, and immediately lysed on ice using the lysis buffer supplied in the RNAqueous RNA isolation kit (Ambion, Austin, TX). Total RNA was isolated with the RNAqueous kit (Ambion, Austin, TX) according to the manufacturer's instructions, and quantified by absorbance at 260 nm. Treatments were performed in triplicate wells within each experiment, and analyses were conducted on RNA preparations from three independent experiments. Expression of specific mRNAs was measured by one-step real time RT-PCR as described [114] using the TaqMan EZ RT-PCR kit (PE Applied Biosystems, Foster City, CA) on the ABI Prism 7700 Sequence Detection System (PE Applied Biosystems). Assays for each sample were carried out in duplicate.

The genes selected for qPCR analysis were: Ar, Esr1, Bmp4, Capn6, Cyp7b1, Sfrp4 and Thbs2. These genes were selected based on strength of response in an accompanying microarray study (Taylor et al., in preparation) and relevance to cell growth. In a microarray study conducted using independent cell cultures under the same conditions described here and using the same concentration of estradiol, estradiol enhanced expression of the mRNA transcripts for 181 genes, including genes encoding ERα and AR, and suppressed mRNA expression of 86 genes ($\geq$1.5-fold change, $p \leq 0.05$). The primer/probe set for Ar was designed using Primer Express software (PE Applied Biosystems), as described [110]. Ar primers were synthesized by Invitrogen, and the Ar probe was synthesized by Applied Biosystems. The concentrations of Mn^{2+}, probe and primers were optimized for the primer/probe set. Other analyses were performed using validated ABI Taqman Gene Expression assays (Applied Biosystems). The RT-PCR assays for each sample were carried out in duplicate. ABI Taqman Gene Expression assays used for specific transcripts were: Mm00433149_m1 (Esr1), Mm00432087_m1 (Bmp4), Mm00500361_m1 (Capn6), Mm00484157_m1 (Cyp7b1), Mm00840104_m1 (Sfrp4) and Mm00449036_m1 (Thbs2). These primers spanned Esr1 exons 3-4, Bmp4 exons 2-3, Capn6 exons 2-3, Cyp7b1 exons 4-5, Sfrp4 exons 4-5 and Thbs2 exons 1-2. The relative concentrations of specific mRNAs in each sample were normalized to total RNA per well, as described [114]. Normalization to total RNA allowed for comparisons between independent experiments and provided a conservative estimate of relative amounts of each mRNA. Differences between control, estradiol and BPA-treated cells were evaluated using the ANOVA GLM procedure in SAS. Comparisons of mean reciprocals for each dose relative to controls were made using the LS Means Test in SAS. The criterion for statistical significance was $p < 0.05$ (two-tailed).

5.3. Results and discussion

The results of the qPCR analysis for Ar, Bmp4, Capn6, Cyp7b1, Esr1, Sfrp4, and Thbs2 are shown in Fig. 5. The data obtained for cells treated with estradiol were consistent with the microarray expression profiles (Taylor et al., in preparation). The data obtained for cells treated with BPA mostly showed similarities, but not in every case. Whereas Ar, Esr1, Cyp7b1 and Sfrp4 were similarly up-regulated and Capn6 was similarly down-regulated by estradiol and BPA, Thbs2 activity was not induced and Bmp4 expression was not inhibited by BPA treatment.

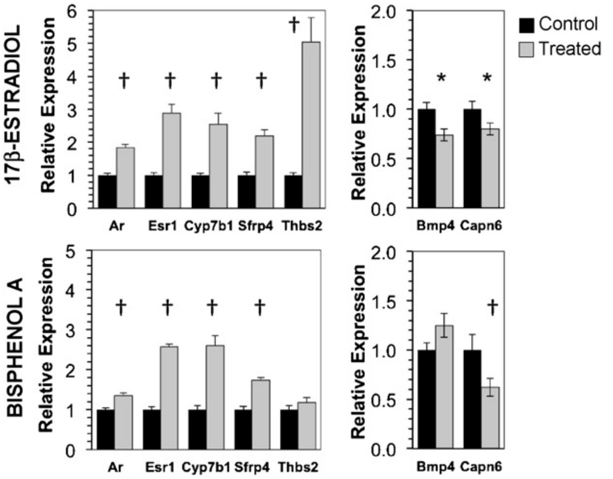

Fig. 5. Expression of selected genes in primary cultures of fetal mouse prostate mesenchyme cells. Effects of E2 (100 nM) or BPA (1000 nM) on expression of seven genes quantified by real-time PCR. Of the seven genes represented, up-regulated genes are shown on the left, down-regulated genes on the right. *$p < 0.05$ or †$p < 0.01$, significantly different compared with control (vehicle-treated) cells.

Neonatal estrogen treatment is known to affect the expression of several genes critical to prostate development, although in our microarray study not all were found to differ in response to treatment with estradiol. There may be several reasons for this, but two are critical. First, we deliberately cultured only the mesenchyme cells to specifically examine effects of estradiol on gene expression in the cells that express ERα and initiate early prostate differentiation. Without the two-way communication that occurs between epithelial and mesenchymal cells in the developing prostate [3,115,116] the full range of gene expression cannot not be determined. For example, Nkx3.1 is expressed only in epithelial cells in regions of ductal growth, although its expression is dependent on the presence of UGS mesenchyme [117]. Similarly, components of the Shh signaling pathway that are important for directing ductal growth are expressed in the mesenchyme but are regulated by Shh signaling from the epithelium [118]. In studies performed *in vivo*, other factors provided via blood circulation can influence gene expression [112], and as a result, studies performed in whole tissues of intact animals are bound to yield different and more complex results. The second reason for a lack of effect on potential candidate genes is that the treatment doses used, which were based on earlier qPCR data for both estradiol and BPA [110], may not have been optimal for each of the genes in question [109].

For example, the data obtained for mesenchyme cells treated with BPA were mostly similar to those from cells treated with estradiol, but not in every case. As noted above, while Ar, Esr1, Cyp7b1 and Sfrp4 were similarly up-regulated and Capn6 was similarly down-regulated by estradiol and BPA, Thbs2 activity was not induced by BPA treatment, and Bmp4 expression was not inhibited. Further, the degree of Ar induction was lower in BPA-treated cells than in estradiol-treated cells. However, the almost identical responses of Esr1 and Cyp7b1 indicate that the relative doses of estradiol and BPA chosen were appropriate for these genes. Shioda et al. have shown that gene expression profiles for different estrogenic chemicals do not always agree and are highly dependent on dose [109]. A complication is thus that different genes have different sensitivities, different-shaped dose–response curves, and different time courses [94,108].

The changes in AR mRNA levels that we observed in cells exposed to estradiol and BPA were between about 1.5-fold for BPA

to 2-fold for estradiol. However, androgen-binding measurements both *in vivo* and in vitro have revealed consistently greater changes, from two-fold to seven-fold, in response to physiological doses of estrogens [16,119]. Thus, the magnitude of change in mRNA levels is not always indicative of the magnitude of change in the physiology of the tissue or organism under study.

Our data indicate that estradiol treatment of fetal prostatic mesenchyme cells has significant effects on genes with cellular functions relevant to steroid hormone signaling and metabolism and to inter- and intra-cellular communications that promote cell growth. Many genes are components of multiple signaling pathways. For example, Bmp4, which was down-regulated by estradiol in this experiment is a component not only of the TGF-β pathway, but also prostate cancer and Shh signaling pathways, and the basal cell carcinoma pathway [120]. The same is true for Wnt11, which we did not test here but was significantly down-regulated by the same dose of estradiol in a related study of mesenchymal gene expression by microarray analysis (Taylor et al., in preparation). Wnt11 acts to inhibit β-catenin stabilization [121,122], and β-catenin has been shown to enhance Ar transcriptional activation by both androgens and estradiol [123]. Again, our culture system only contains UGS mesenchymal cells and thus the full range of effects on specific signaling pathways cannot be determined. Within the TGF-β signaling pathway we observed up-regulation of Thbs2 by estradiol but not BPA; Thbs2 is a negative regulator of Bmp4 activity [124]. Taken together, these results are consistent and suggest that the TGF-β signaling pathway is suppressed by estradiol treatment, which is associated with an increase in epithelial proliferation that we observed in the dorsolateral prostate of male mouse fetuses in response to estrogen (DES, BPA and ethinylestradiol) administration to pregnant mice [74].

While we observed in our microarray analysis that estradiol led to down-regulation of Wnt11, there was significant up-regulation of Wnt7b. As discussed, Wnt11 signals through non-canonical Wnt signaling pathways, which inhibit β-catenin stabilization, while Wnt7b signals through the canonical Wnt/β-catenin stabilization pathway [125]. Secreted Frizzled related proteins (SFRPs) are a family of secreted proteins that can bind to frizzled receptors and also to Wnt ligands, thus interfering with Wnt signaling. However, different members of the SFRP family have markedly different activities [126]. SFRPs that favor cell death diminish β-catenin stability, whereas SFRPs that promote β-catenin accumulation increase cell resistance to apoptosis induced by various agents. The observed up-regulation of a member of this gene family, Sfrp4, is consistent with up-regulation of the canonical Wnt signaling pathway and epithelial proliferation. Finally, Capn6, one of a family of calcium-dependent cysteine proteases that are implicated in apoptosis, was also down-regulated by both estradiol and BPA, suggesting inhibitory effects of estrogen on cell death.

In addition to effects on steroid receptor mRNA expression, estrogen treatment also altered the expression of genes important for steroid hormone metabolism in these cells. Specifically, here we showed that both estradiol and BPA up-regulated Cyp7b1, a critical factor in DHT metabolism [127] that metabolizes the DHT product 3β-androstanediol. Interestingly, 3β-androstanediol has been identified as a ligand for ERβ and a negative regulator of ventral prostate growth [128]. Upregulation of Cyp7b1 in this experiment is thus consistent with stimulation of cell growth.

Other inter-cellular signals are implicated in prostate differentiation and subsequent growth. For example, EGF is likely to mediate at least some of the stimulatory effects of estrogenic chemicals on initial prostate budding and thus the number of prostate glands, while both EGF and IGF-1, as well as other growth factors [3] [2], mediate effects of estrogenic chemicals on growth of prostatic glandular epithelium and branching morphogenesis. For example, in one study Gupta reported that EGF was required for DES-induced growth and branching of mouse prostate organ cultures, while IGF-1 was required only for DES-induced branching morphogenesis [119].

6. Controversies concerning the stimulating effect of estradiol, bisphenol A and estrogenic drugs on the prostate in mice

There are relatively few studies that have reported no effects of developmental BPA exposure on the mouse prostate or other outcomes, and the majority of these "no effect" studies have been funded by the chemical industry [29,129,130]. For example, one study [131] reported that exposure of fetal mice to low doses of either BPA or the positive control, DES, produced no effect on the development of the prostate. Another study [132] reported no effects of low doses of BPA on the prostate, although a response in their experiment required an extremely high dose of the positive control estradiol (100 μg/kg/day). This chemical-industry funded study by Tyl et al. has been used by regulatory agencies to declare that BPA is safe because it was conducted using a data reporting system named Good Laboratory Practices or GLP [133,134]. It is interesting that in both of these studies that did not find any effects of low doses of BPA, the animals were maintained on Purina soy-based 5002 feed, which appears to contain ingredients that disrupt normal development of the systems that are impacted by BPA [113]. These results suggest that some component of the diet caused a maximal increase in prostate size in control males in the study, and no further increase in size in response to either DES or BPA could then be observed. Our findings with estradiol and DES [16] show that there is a maximum amount of increase in adult prostate size due to prenatal exposure to estrogenic chemicals, after which an increase in dose results in a decrease in prostate size, forming an inverted-U dose response curve.

It is not surprising that in addition to estrogenic drugs, estrogenic pesticides and estrogenic plastic monomers and antioxidants, there are components of formulated laboratory animal diets that can modulate the development of the reproductive system and change postnatal growth rate and reproductive organs, including the prostate [135–137]. There is now evidence that phytoestrogens such as genistein (one of the primary phytoestrogens in soy) interact differently with estrogen response systems relative to BPA [26,138]. The animal feed we use in our research results in lean control animals without enlarged prostates, and effects of very low doses of estrogenic chemicals on the development of accessory reproductive organs in males maintained on this diet have been consistently observed with numerous estrogenic chemicals, such as estradiol, DES, ethinylestradiol, bisphenol A and methoxychlor, in experiments conducted over many years (e.g. Figs. 3 and 6).

A recent study examined the effects of perinatal exposure (via the mother) to ethinylestradiol and BPA on development of the male reproductive organs in Long-Evans rats. In this study an ethinylestradiol dose of 50 μg/kg/day was required to result in a significant decrease in ventral prostate weight in the male offspring when examined in adulthood [139], but no effect of BPA was seen. The highest dose of BPA administered in this study was 200 μg/kg/day, only 4-fold higher than the dose of ethinylestradiol dose required to alter prostate development. Because ethinylestradiol is typically at least 100-fold more potent than BPA it is likely that the BPA dose range chosen here (based on mouse exposure studies) was not wide enough establish the effective dose in this animal model, a fact that the authors acknowledged, and thus no effect on prostate development at this dose of BPA would be expected. The authors also discussed the fact that different strains of rat have widely differing sensitivities to estrogenic and antiestrogenic chemicals, which tend to vary by trait rather than in terms of a general sensitivity (or lack of it) to estrogens. This thus

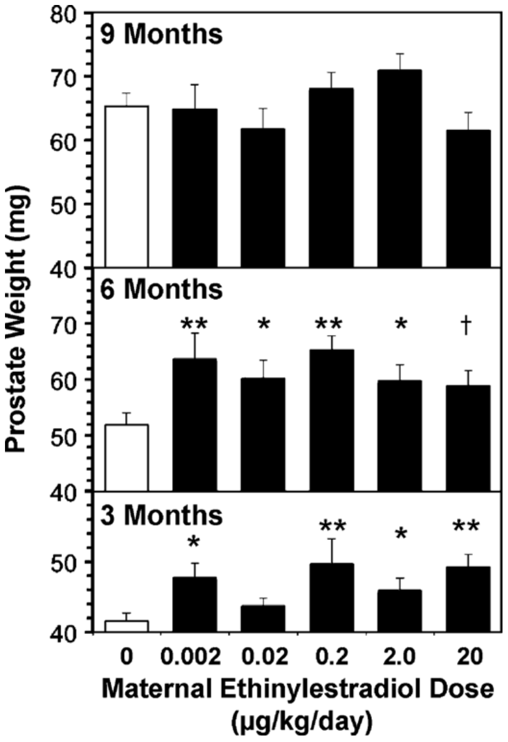

Fig. 6. Prostate weight at different ages in CD-1 male offspring of females fed ethinylestradiol at a dose of 0.002–20 μg/kg/day in tocopherol-stripped corn oil from GD 14-18; controls (0 dose) were fed just oil. Males were examined at 3, 6 and 9 months of age. For both control and EE-treated males, there was an age-related increase in prostate size. However, at 3 and 6 months of age, ethinylestradiol-treated males had larger prostates relative to controls of the same age. $^{*}p < 0.05$, $^{**}p < 0.01$, † $p = 0.06$ vs. controls of the same age.

also emphasizes the need to consider species and strain differences when assessing results of chemical exposures.

In the study discussed above by Tyl [132], no effects of BPA on the prostate of CD-1 mice were found at any dose, but the dose of the positive control, estradiol, required to cause an effect was 100 μg/kg/day. The prostates in the control CD-1 mice from this experiment were reported in the initial article [132] to weigh (mean ± SEM) 74.4 ± 2.9 mg at 14 weeks old, which is an abnormally high prostate weight for this age (body weight was 40.46 ± 0.67 g). However, it is difficult to assess these data because subsequently these same animals were identified as having been examined when 18 weeks old [140], and at a FDA hearing (September 16, 2008) in Rockville MD, Tyl presented testimony that the same animals were 24 weeks old. Given that this study was conducted under GLP guidelines, the reporting by the senior author of different data in multiple fora from a study being used to assess the risk of BPA by regulatory agencies is highly unusual.

For many years the common argument was that BPA was a weak estrogen and could not possibly cause effects at doses lower than 5000 μg/kg/day, a dose that remains the predicted no effect level (NOEL) according to US and European regulatory agencies [141,142]. The large number of studies showing effects of BPA in human and animal cells at doses within and below the picogram per ml (pM) range provide ample evidence that BPA is not a weak estrogenic chemical for many responses [30,69]. However, the finding that a dose of BPA below the current daily intake dose declared safe for humans throughout the lifetime by the EPA [142] increases aromatase activity and intracellular estradiol levels in fetal prostate mesenchyme by almost 20 parts per trillion [112] adds another layer of complexity to this debate. These findings by Arase and colleagues provide evidence that BPA can cause unexpected effects that can result in changes in endocrine function that without question can account for intracellular changes at very low, presumably completely safe, doses. There can be no argument that an intracellular increase in estradiol of 20 parts per trillion is a physiologically active concentration of estradiol. In fact, in both the fetal prostate mesenchyme [16,110] and MCF-7 cells [47], estradiol alters gene activity and stimulates proliferation at a dose of 0.28 pg/ml (1 pM). The rodent and human male fetus circulates testosterone at nM concentrations during sexual differentiation [16,143], which provides high concentrations of substrate for the aromatization to estradiol in fetal tissues.

7. Experiment to examine effects of ethinylestradiol administration to pregnant CD-1 mice: significant enlargement of the prostate in adult male offspring at subclinical doses

7.1. Introduction

Here we report the results from an experiment in which we examined the effect of fetal exposure to ethinylestradiol in CD-1 mice on prostate size at different ages. In a prior study we found control 3-month-old CD-1 males to have body weights of about 40 g and prostate weights of about 42 mg when fed our combination of standard soy-based Purina 5008/5001 feeds [137]. These data were very similar to those from a study by Heindel et al. [144], in which the mean prostate weight of 16–17-week-old CD-1 male mice was reported to be 45.9 mg. The study by Heindel et al. [144] was conducted in the same institute that Tyl's 2008 GLP study was conducted in (Research Triangle Institute), but with very different results for control animals.

The objective of our study was to expose mouse fetuses to ethinylestradiol during the initial period of prostate differentiation during fetal life. Use of oral contraceptives in unrecognized pregnancy has been reported to continue well into the first trimester and may extend into the 16th week of gestation and is associated with abnormalities of the urogenital system [145]. Since women do not expect to be pregnant, exposure to ethinylestradiol throughout the initial period of accessory reproductive organ differentiation in human fetuses is not unusual. Sexual differentiation begins at the end of the second month of gestation in humans, and prostate differentiation begins during week 10. Prostate differentiation begins on gestation day 17 in mice, and at birth, mice are at approximately the equivalent of the gestation week 17-stage in humans. The neonatal period of continued prostate differentiation (branching morphogenesis) in mice occurs during fetal life in humans [12].

7.2. Materials and methods

Pregnant CD-1 mice were fed (in tocopherol-stripped corn oil) doses of ethinylestradiol that ranged from approximately 100-fold lower than doses in oral contraceptives (per kg body weight) to doses up to about 200-fold greater than the clinically relevant dose (approximately 0.3–0.5 μg/kg, depending on body weight and dose). Between gestation day 14–18 pregnant female CD-1 mice were fed ethinylestradiol at doses of 0.002, 0.02, 0.2, 2 and 20 μg/kg/day. Gestation day 18 is roughly equivalent to approximately gestation week 15–16 in humans. We had previously reported that these maternal doses of ethinylestradiol resulted in significantly enlarged prostates in another strain of mice (CF-1) when the male offspring were examined in young adulthood [146].

7.3. Results and discussion

We observed an age-related increase in prostate size in control males through 9 months of age. However, at 3 and 6 months old, males exposed ethinylestradiol during fetal life (including

those exposed to the lowest 0.002 μg/kg/day dose) had significantly enlarged prostates relative to controls (Fig. 6), confirming prior findings in CF-1 mice at about two-months of age [146]. Although there were age-dependent increases in body weight in all treatment groups (data not shown), body weight was not different as a function of treatment. Body weight and prostate weight were not correlated within the 3 and 6-month age groups, although there was a significant correlation at 9 months of age [147].

In this study at both 3 and 6 months of age, male CD-1 mice exposed as fetuses to ethinylestradiol showed enlarged prostates as a result of the dam ingesting a 0.002 μg/kg/day dose, which is more than 100-fold lower than the clinical dose of ethinylestradiol in oral contraceptives [146]. This finding demonstrates that the increase in prostate size caused by brief fetal exposure to very low doses of ethinylestradiol previously identified in CD-1 male fetuses [74] results in an enlarged prostate into young adulthood, replicating the prior finding in CD-1 mice by Gupta using very low doses of DES and BPA using the same method and time of fetal exposure [14]. However, by the time males were 9 months old, the prostates of control animals had reached a mean (±SEM) size of 65.3 ± 2.1 mg, an increase in size that we previously observed to occur between 9 and 12 months of age in CF-1 mice [148].

While it remains unclear at what age the CD-1 males in the study conducted by Tyl et al. [140] were examined, our findings indicate that whether they were 14, 18 or 24 weeks old, if the prostate weights for control animals were on average greater than 70 mg as reported [132,140], these control males were clearly abnormal, and the grossly enlarged prostates in these relatively young males would have masked any significant differences due to treatment with estrogenic chemicals. These findings indicate that regulatory agencies should reconsider using the data from the study by Tyl and colleagues [132], since it appears that at the time the organs were examined, the control animals were markedly different from historic values, even in relation to data for CD-1 male mice from a prior study conducted at the same institute [144].

8. Conclusions

For many years estrogen was thought to inhibit prostate development because estrogenic drugs such as DES, administered at very high, supra-physiological doses, interfered with normal prostate development. However, there is now extensive evidence that at very low concentrations, exposure during the fetal and neonatal period of prostate differentiation in rodents to endogenous estradiol, estrogenic drugs, and environmental chemicals with estrogenic activity can stimulate prostate growth and lead to prostate disease in later adulthood [6]. Studies are beginning to identify the signaling systems that mediate these effects, but much remains to be determined regarding the mechanisms in experimental laboratory animals. Results of research presented here in which we examined a number of candidate genes in the fetal mouse prostate mesenchyme that are involved in proliferation and apoptosis, were consistent with estradiol and the estrogenic chemical BPA stimulating proliferation of prostate epithelium in male mouse fetuses. We previously detected hyperplasia of basal epithelial cells in the primary prostatic ducts *in vivo* at very low doses of BPA, DES and ethinylestradiol administered to pregnant mice [74].

There is relatively little known about the developmental effects on estrogenic chemicals on the prostate in human fetuses. The DES sons (male offspring exposed in utero to DES) were exposed to very high doses of DES, and they are thus unlikely to show effects that are predictive of exposure to low doses of environmental estrogens such as BPA or to ethinylestradiol in oral contraceptives. The medical community has been unaware that there may be health consequences for the millions of offspring produced each year by women who become pregnant while using oral contraceptives containing ethinylestradiol, largely because the exposed offspring do not show external malformations at birth [146]. This is reminiscent of the DES tragedy, where gestational use of DES continued for decades because a lack of outward abnormality in the newborn was taken to imply no harm; damage to the reproductive tract of abortuses was confirmed histologically almost ten years after DES was banned for use in pregnancy [149]. As we have shown in research presented here and previously, abnormal development of the male reproductive system is clearly visible in male mouse fetuses at subclinical doses of ethinylestradiol and at doses of BPA declared to be safe by regulatory agencies [74]. The finding that exposure to estrogen later in adulthood, which occurs as a natural event during aging in men [150], results in the development of high grade prostate intraepithelial neoplastic (HG-PIN) lesions in male rats exposed during development to low doses of estradiol or BPA [97] indicates that there should be high concern that a similar outcome could occur during aging in men. Given that the “plastic revolution” began in the 1970s, the cohort of men exposed to high levels of environmental chemicals such as BPA has not reached the age at which pathology of the prostate would be expected. This issue might be clarified by the investigation of the long-term consequences for male or female offspring of women who continued to take oral contraceptives through the period of pregnancy when differentiation of the reproductive organs occurs.

The findings presented here provide additional evidence that the environmental estrogenic chemical, BPA, disrupts prostate development in male mice by stimulating genes in mesenchyme cells that are involved in epithelial proliferation and inhibiting genes involved in apoptosis. BPA also disrupts development of the entire male reproductive system in mice [17]. Additionally, there are a wide range of other adverse effects on males and females exposed during development to doses of BPA below the current EPA reference dose of 50 ug/kg/day [29,41], which is predicted by the EPA to be a safe lifetime exposure level [29,41].

Disclosure statement

JAT, KAR and RLR have nothing to disclose. FSvS has been a consultant for attorneys and written reports reviewing the published literature about the health effects of BPA in animals and humans.

Acknowledgement

This work was supported by a grant from NIH to FVS (ES018764).

References

[1] G.R. Cunha, W.A. Ricke, A. Thomson, P.C. Marker, G. Risbridger, S.W. Hayward, Y.Z. Wang, A.A. Donjacour, T. Kurita, Hormonal, cellular, and molecular regulation of normal and neoplastic prostatic development, J. Steroid Biochem. Mol. Biol. 92 (4) (2004) 221–236.

[2] G.S Prins, O. Putz, Molecular signaling pathways that regulate prostate gland development, Differentiation 76 (6) (2008) 641–659.

[3] P.C. Marker, A.A. Donjacour, R. Dahiya, G.R. Cunha, Hormonal, cellular, and molecular control of prostatic development, Develop. Biol. 253 (2) (2003) 165–174.

[4] P.V Nantermet, J. Xu, Y. Yu, P. Hodor, D. Holder, S. Adamski, M.A. Gentile, D.B. Kimmel, S. Harada, D. Gerhold, L.P. Freedman, W.J. Ray, Identification of genetic pathways activated by the androgen receptor during the induction of proliferation in the ventral prostate gland, J. Biol. Chem. 279 (2) (2004) 1310–1322.

[5] D.S. Coffey, K.J. Pienta, New concepts in studying the control of normal and cancer growth of the prostate, in: D.S. Coffey, A. Chiarodo, J.P. Karr (Eds.), Current Concepts and Approaches to the Study of Prostate Cancer, Alan R. Liss, 1987, pp. 1–73.

[6] G.S. Prins, L. Birch, W.Y. Tang, S.M. Ho, Developmental estrogen exposures predispose to prostate carcinogenesis with aging, Reprod. Toxicol. 23 (3) (2007) 374–382.

[7] W.A. Ricke, S.J. McPherson, J.J. Bianco, G.R. Cunha, Y. Wang, G.P. Risbridger, Prostatic hormonal carcinogenesis is mediated by in situ estrogen production and estrogen receptor alpha signaling, FASEB J. 22 (5) (2008) 1512–1520.
[8] G. Risbridger, H. Wang, P. Young, T. Kurita, Y.Z. Wang, D. Lubahn, J.A. Gustafsson, G. Cunha, Evidence that epithelial and mesenchymal estrogen receptor-alpha mediates effects of estrogen on prostatic epithelium, Dev. Biol. 229 (2) (2001) 432–442.
[9] G.P. Risbridger, H. Wang, M. Frydenberg, G.R. Cunha, The metaplastic effects of estrogen on mouse prostate epithelium: Proliferation of cells with basal cell phenotype, Endocrinology 142 (6) (2001) 2443–2450.
[10] P. Martikainen, S. Makela, R. Santti, P. Harkonen, J. Souminen, Interaction of male and female sex hormones in cultured rat prostate, Prostate 11 (1987) 291–303.
[11] G.S. Prins, K.S. Korach, The role of estrogens and estrogen receptors in normal prostate growth and disease, Steroids 73 (3) (2008) 233–244.
[12] C.A. Richter, B.G. Timms, F.S. vom Saal, Prostate development: mechanisms for opposite effects of low and high doses of estrogenic chemicals, in: R.K. Naz (Ed.), Endocrine Disruptors: Effects on Male and Female Reproductive Systems., CRC Press, Boca Raton, FL, 2005, pp. 379–410.
[13] G.R. Cunha, A. Donjacour, P. Cooke, S. Mee, R. Bigsby, S. Higgins, Y. Sugimura, The endocrinology and developmental biology of the prostate, Endocrine Rev. 8 (1987) 338–362.
[14] C. Gupta, Reproductive malformation of the male offspring following maternal exposure to estrogenic chemicals, Proc. Soc. Exp. Biol. Med. 224 (2) (2000) 61–68.
[15] S.C. Nagel, F.S. vom Saal, K.A. Thayer, M.G. Dhar, M. Boechler, W.V. Welshons, Relative binding affinity-serum modified access (RBA-SMA) assay predicts the relative in vivo bioactivity of the xenoestrogens bisphenol A and octylphenol, Environ. Health Perspect. 105 (1) (1997) 70–76.
[16] F.S. vom Saal, B.G. Timms, M.M. Montano, P. Palanza, K.A. Thayer, S.C. Nagel, M.D. Dhar, V.K. Ganjam, S. Parmigiani, W.V. Welshons, Prostate enlargement in mice due to fetal exposure to low doses of estradiol or diethylstilbestrol and opposite effects at high doses, Proc. Natl. Acad. Sci. U S A 94 (5) (1997) 2056–2061.
[17] F.S. vom Saal, P.S. Cooke, D.L. Buchanan, P. Palanza, K.A. Thayer, S.C. Nagel, S. Parmigiani, W.V. Welshons, A physiologically based approach to the study of bisphenol A and other estrogenic chemicals on the size of reproductive organs, daily sperm production, and behavior, Toxicol. Ind. Health 14 (1–2) (1998) 239–260.
[18] W.V. Welshons, S.C. Nagel, K.A. Thayer, B.M. Judy, F.S. Vom Saal, Low-dose bioactivity of xenoestrogens in animals: fetal exposure to low doses of methoxychlor and other xenoestrogens increases adult prostate size in mice, Toxicol. Ind. Health 15 (1–2) (1999) 12–25.
[19] G.G.J.M. Kuiper, E. Enmark, M. Pelto-Huikko, S. Nilsson, J.-A. Gustafsson, Cloning of a novel estrogen receptor expressed in rat prostate and ovary, Proc. Natl. Acad. Sci. 93 (1996) 5925–5930.
[20] Y. Omoto, O. Imamov, M. Warner, J.A. Gustafsson, Estrogen receptor alpha and imprinting of the neonatal mouse ventral prostate by estrogen, Proc. Natl. Acad. Sci. U S A 102 (5) (2005) 1484–1489.
[21] J.Y. Adams, I. Leav, K.M. Lau, S.M. Ho, S.M. Pflueger, Expression of estrogen receptor beta in the fetal, neonatal, and prepubertal human prostate, Prostate 52 (1) (2002) 69–81.
[22] N. Sugiyama, R.P. Barros, M. Warner, J.A. Gustafsson, ERbeta: recent understanding of estrogen signaling, Trends Endocrinol. Metab. 21 (9) (2010) 545–552.
[23] B.G. Timms, S.L. Petersen, F.S. vom Saal, Prostate gland growth during development is stimulated in both male and female rat fetuses by intrauterine proximity to female fetuses, J. Urol. 161 (1999) 1694–1701.
[24] G.S. Prins, M. Marmer, C. Woodham, W. Chang, G. Kuiper, J.A. Gustafsson, L. Birch, Estrogen receptor-beta messenger ribonucleic acid ontogeny in the prostate of normal and neonatally estrogenized rats, Endocrinology 139 (3) (1998) 874–883.
[25] G.S. Prins, L. Birch, J.F. Couse, I. Choi, B. Katzenellenbogen, K.S. Korach, Estrogen imprinting of the developing prostate gland is mediated through stromal estrogen receptor alpha: studies with alphaERKO and betaERKO mice, Cancer Res. 61 (16) (2001) 6089–6097.
[26] Y.K. Leung, P. Mak, S. Hassan, S.M. Ho, Estrogen receptor (ER)-beta isoforms: a key to understanding ER-beta signaling, Proc. Natl. Acad. Sci. U S A 103 (35) (2006) 13162–13167.
[27] S.J. Ellem, G.P. Risbridger, The dual, opposing roles of estrogen in the prostate, Ann. N. Y. Acad. Sci. 1155 (2009) 174–186.
[28] V.C Jordan, C.S. Murphy, Endocrine pharmacology of antiestrogens as antitumor agents, Endocr. Rev. 11 (4) (1990) 578–610.
[29] C.A. Richter, L.S. Birnbaum, F. Farabollini, R.R. Newbold, B.S. Rubin, C.E. Talsness, J.G. Vandenbergh, D.R. Walser-Kuntz, F.S. vom Saal, In vivo effects of bisphenol A in laboratory rodent studies, Reprod. Toxicol. 24 (2) (2007) 199–224.
[30] W.V. Welshons, S.C. Nagel, F.S. vom Saal, Large effects from small exposures. III. Endocrine mechanisms mediating effects of bisphenol A at levels of human exposure, Endocrinology 147 (6 Suppl.) (2006) S56–S69.
[31] E.J. Routledge, R. White, M.G. Parker, J.P. Sumpter, Differential effects of xenoestrogens on coactivator recruitment by estrogen receptor (ER) alpha and ERbeta, J. Biol. Chem. 275 (46) (2000) 35986–35993.
[32] C.L. Smith, B.W. O'Malley, Coregulator function: a key to understanding tissue specificity of selective receptor modulators, Endocr. Rev. 25 (1) (2004) 45–71.
[33] G.G. Kuiper, B. Carlsson, K. Grandien, E. Enmark, J. Haggblad, S. Nilsson, J.A. Gustafsson, Comparison of the ligand binding specificity and transcript tissue distribution of estrogen receptors alpha and beta, Endocrinology 138 (3) (1997) 863–870.
[34] T. Hajszan, C. Leranth, Bisphenol A interferes with synaptic remodeling, Front. Neuroendocrinol. 31 (4) (2010) 519–530.
[35] C. Leranth, T. Hajszan, K. Szigeti-Buck, J. Bober, N.J. MacLusky, Bisphenol A prevents the synaptogenic response to estradiol in hippocampus and prefrontal cortex of ovariectomized nonhuman primates, Proc. Natl. Acad. Sci. U S A 105 (37) (2008) 14187–14191.
[36] N.J. MacLusky, T. Hajszan, C. Leranth, The environmental estrogen bisphenol A inhibits estrogen-induced hippocampal synaptogenesis, Environ. Health Perspect. 113 (2005) 675–679.
[37] R.A. Alyea, C.S. Watson, Differential regulation of dopamine transporter function and location by low concentrations of environmental estrogens and 17beta-estradiol, Environ. Health Perspect. 117 (5) (2009) 778–783.
[38] A. Zsarnovszky, H.H. Le, H.S. Wang, S.M. Belcher, Ontogeny of rapid estrogen-mediated extracellular signal-regulated kinase signaling in the rat cerebellar cortex: potent nongenomic agonist and endocrine disrupting activity of the xenoestrogen bisphenol A, Endocrinology 146 (12) (2005) 5388–5396.
[39] C.S. Watson, N.N. Bulayeva, A.L. Wozniak, R.A. Alyea, Xenoestrogens are potent activators of nongenomic estrogenic responses, Steroids 72 (2) (2007) 124–134.
[40] Y.J. Jeng, M.Y. Kochukov, C.S. Watson, Membrane estrogen receptor-alpha-mediated nongenomic actions of phytoestrogens in GH3/B6/F10 pituitary tumor cells, J. Mol. Signal. 4 (2009) 2.
[41] F.S. vom Saal, B.T. Akingbemi, S.M. Belcher, L.S. Birnbaum, D.A. Crain, M. Eriksen, F. Farabollini, L.J. Guillette Jr., R. Hauser, J.J. Heindel, S.M. Ho, P.A. Hunt, T. Iguchi, S. Jobling, J. Kanno, R.A. Keri, K.E. Knudsen, H. Laufer, G.A. LeBlanc, M. Marcus, J.A. McLachlan, J.P. Myers, A. Nadal, R.R. Newbold, N. Olea, G.S. Prins, C.A. Richter, B.S. Rubin, C. Sonnenschein, A.M. Soto, C.E. Talsness, J.G. Vandenbergh, L.N. Vandenberg, D.R. Walser-Kuntz, C.S. Watson, W.V. Welshons, Y. Wetherill, R.T. Zoeller, Chapel Hill bisphenol A expert panel consensus statement: integration of mechanisms, effects in animals and potential to impact human health at current levels of exposure, Reprod. Toxicol. 24 (2) (2007) 131–138.
[42] T. Fujimura, S. Takahashi, T. Urano, N. Ijichi, K. Ikeda, J. Kumagai, T. Murata, K. Takayama, K. Horie-Inoue, Y. Ouchi, M. Muramatsu, Y. Homma, S. Inoue, Differential expression of estrogen-related receptors beta and gamma (ERRbeta and ERRgamma) and their clinical significance in human prostate cancer, Cancer Sci. 101 (3) (2010) 646–651.
[43] S. Yu, X. Wang, C.F. Ng, S. Chen, F.L. Chan, ERRgamma suppresses cell proliferation and tumor growth of androgen-sensitive and androgen-insensitive prostate cancer cells and its implication as a therapeutic target for prostate cancer, Cancer Res. 67 (10) (2007) 4904–4914.
[44] T. Nose, Y. Shimohigashi, A docking modelling rationally predicts strong binding of bisphenol A to estrogen-related receptor gamma, Protein Pept. Lett. 15 (3) (2008) 290–296.
[45] H. Okada, T. Tokunaga, X. Liu, S. Takayanagi, A. Matsushima, Y. Shimohigashi, Direct evidence revealing structural elements essential for the high binding ability of bisphenol A to human estrogen-related receptor-gamma, Environ. Health Perspect. 116 (1) (2008) 32–38.
[46] L.N. Vandenberg, I. Chahoud, J.J. Heindel, V. Padmanabhan, F.J. Paumgartten, G. Schoenfelder, Urinary. circulating, and tissue biomonitoring studies indicate widespread exposure to bisphenol A, Environ. Health Perspect. 118 (8) (2010) 1055–1070.
[47] W.V. Welshons, K.A. Thayer, B.M. Judy, J.A. Taylor, E.M. Curran, F.S. vom Saal, Large effects from small exposures. I. Mechanisms for endocrine-disrupting chemicals with estrogenic activity, Environ. Health Perspect. 111 (8) (2003) 994–1006.
[48] H.J. Lee, S. Chattopadhyay, E.Y. Gong, R.S. Ahn, K. Lee, Antiandrogenic effects of bisphenol A and nonylphenol on the function of androgen receptor, Toxicol. Sci. 75 (1) (2003) 40–46.
[49] F. Paris, P. Balaguer, B. Terouanne, N. Servant, C. Lacoste, J.P. Cravedi, J.C. Nicolas, C. Sultan, Phenylphenols, biphenols, bisphenol-A and 4-tert-octylphenol exhibit alpha and beta estrogen activities and antiandrogen activity in reporter cell lines, Mol. Cell Endocrinol. 193 (1–2) (2002) 43–49.
[50] S. Yeh, H. Miyamato, H. Shima, C. Chang, From estrogen to androgen receptor: a new pathway for sex hormones in prostate, Proc. Natl. Acad. Sci. 95 (1998) 5527–5532.
[51] A. Ishihara, N. Nishiyama, S. Sugiyama, K. Yamauchi, The effect of endocrine disrupting chemicals on thyroid hormone binding to Japanese quail transthyretin and thyroid hormone receptor, Gen. Comp. Endocrinol. 134 (1) (2003) 36–43.
[52] K. Moriyama, T. Tagami, T. Akamizu, T. Usui, M. Saijo, N. Kanamoto, Y. Hataya, A. Shimatsu, H. Kuzuya, K. Nakao, Thyroid hormone action is disrupted by bisphenol A as an antagonist, J. Clin. Endocrinol. Metab. 87 (11) (2002) 5185–5190.
[53] J.K. Hess-Wilson, S.L. Webb, H.K. Daly, Y.K. Leung, J. Boldison, C.E. Comstock, M.A. Sartor, S.M. Ho, K.E. Knudsen, Unique bisphenol A transcriptome in prostate cancer: novel effects on ERbeta expression that correspond to androgen receptor mutation status, Environ. Health Perspect. 115 (11) (2007) 1646–1653.
[54] G.G. Kuiper, J.G. Lemmen, B. Carlsson, J.C. Corton, S.H. Safe, P.T. van der Saag, B. van der Burg, J.A. Gustafsson, Interaction of estrogenic chemicals and

phytoestrogens with estrogen receptor beta, Endocrinology 139 (10) (1998) 4252–4263.
[55] E.C. Dodds, W. Lawson, Synthetic oestrogenic agents without the phenanthrene nucleus, Nature 137 (1936) 996.
[56] M. Kissinger, FDA says it's unable to regulate BPA, Milwaukee J. Sentinel (2010), Milwaukee, http://www.jsonline.com/watchdog/watchdogreports/81901927.html.
[57] P.D. Bailin, M. Byrne, S. Lewis, R. Liroff, Public awareness drives market for safer alternatives: bisphenol A market analysis report. http://www.iehn.org/publications.reports.bpa.php. Accessed date: May 30, 2010, 2008.
[58] K.L. Howdeshell, P.H. Peterman, B.M. Judy, J.A. Taylor, C.E. Orazio, R.L. Ruhlen, F.S. Vom Saal, W.V. Welshons, Bisphenol A is released from used polycarbonate animal cages into water at room temperature, Environ. Health Perspect. 111 (9) (2003) 1180–1187.
[59] J.A. Taylor, F.S. vom Saal, W.V. Welshons, B. Drury, G. Rottinghaus, P.A. Hunt, C.A. Vandevoort, Similarity of bisphenol A pharmacokinetics in rhesus monkeys and mice: relevance for human exposure, Environ. Health Perspect. 119 (2011) 422–430, Online DOI: 10.1289/ehp.1002514.
[60] L.N. Vandenberg, R. Hauser, M. Marcus, N. Olea, W.V. Welshons, Human exposure to bisphenol A (BPA), Reprod. Toxicol. 24 (2) (2007) 139–177.
[61] A.M. Calafat, X. Ye, L.Y. Wong, J.A. Reidy, L.L. Needham, Exposure of the U.S. population to bisphenol A and 4-tertiary-octylphenol: 2003–2004, Environ. Health Perspect. 116 (1) (2008) 39–44.
[62] L.N. Vandenberg, I. Chahoud, V. Padmanabhan, F.J. Paumgartten, G. Schoenfelder, Biomonitoring studies should be used by regulatory agencies to assess human exposure levels and safety of bisphenol A, Environ. Health Perspect. 118 (8) (2010) 1051–1054.
[63] J.M. Braun, K. Yolton, K.N. Dietrich, R. Hornung, X. Ye, A.M. Calafat, B.P. Lanphear, Prenatal bisphenol A exposure and early childhood behavior, Environ. Health Perspect. 117 (2009) 1945–1952.
[64] V.Y. Fujimoto, D. Kim, F.S. Vom Saal, J.D. Lamb, J.A. Taylor, M.S. Bloom, Serum unconjugated bisphenol A concentrations in women may adversely influence oocyte quality during in vitro fertilization, Fertil Steril. 95 (5) (2011) 1816–1819.
[65] I.A. Lang, T.S. Galloway, A. Scarlett, W.E. Henley, M. Depledge, R.B. Wallace, D. Melzer, Association of urinary bisphenol A concentration with medical disorders and laboratory abnormalities in adults, JAMA 300 (11) (2008) 1303–1310.
[66] J.D. Meeker, A.M. Calafat, R. Hauser, Urinary bisphenol A concentrations in relation to serum thyroid and reproductive hormone levels in men from an infertility clinic, Environ. Sci. Technol. 44 (4) (2010) 1458–1463.
[67] J.D. Meeker, S. Ehrlich, T.L. Toth, D.L. Wright, A.M. Calafat, A.T. Trisini, X. Ye, R. Hauser, Semen quality and sperm DNA damage in relation to urinary bisphenol A among men from an infertility clinic, Reprod. Toxicol. 30 (4) (2010) 532–539.
[68] D. Melzer, N.E. Rice, C. Lewis, W.E. Henley, T.S. Galloway, Association of urinary bisphenol a concentration with heart disease: evidence from NHANES 2003/06, PLoS One 5 (1) (2010) e8673.
[69] C.S. Watson, Y.J. Jeng, M.Y. Kochukov, Nongenomic signaling pathways of estrogen toxicity, Toxicol. Sci. 115 (1) (2010) 1–11.
[70] R. Newbold, Cellular and molecular effects of developmental exposure to diethylstilbestrol: implications for other environmental estrogens, Environ. Health Perspect. 103 (Suppl 7) (1995) 83–87.
[71] S.H. Swan, F.S. vom Saal, Alterations in male reproductive development: the role of endocrine disrupting chemicals, in: M. Metzler (Ed.), Endocrine Disruptors in the Environment, Springer Verlag, Heidelberg, 2001, pp. p131–p170.
[72] R.W. Stahlhut, W.V. Welshons, S.H. Swan, Bisphenol A data in NHANES suggest longer than expected half-life, substantial nonfood exposure, or both, Environ. Health Perspect. 117 (5) (2009) 784–789.
[73] Y. Ogura, K. Ishii, H. Kanda, M. Kanai, K. Arima, Y. Wang, Y. Sugimura, Bisphenol A induces permanent squamous change in mouse prostatic epithelium, Differentiation Res. Biol. Diversity 75 (8) (2007) 745–756.
[74] B.G. Timms, K.L. Howdeshell, L. Barton, S. Bradley, C.A. Richter, F.S. vom Saal, Estrogenic chemicals in plastic and oral contraceptives disrupt development of the mouse prostate and urethra, Proc. Natl. Acad. Sci. 102 (2005) 7014–7019.
[75] X. Long, R. Steinmetz, N. Ben-Jonathan, A. caperell-Grant, P.C.M. Young, K.P. Nephew, R.M. Bigsby, Strain differencesi in vaginal responses to the xenoestrogen bisphenol A, Environ. Health Perspect. 108 (2000) 243–247.
[76] C.M. Markey, C.L. Michaelson, E.C. Veson, C. Sonnenschein, A.M. Soto, The mouse uterotrophic assay: a reevaluation of its validity in assessing the estrogenicity of bisphenol A, Environ. Health Perspect. 109 (1) (2001) 55–60.
[77] S.C. Nagel, F.S. vom Saal, W.V. Welshons, The effective free fraction of estradiol and xenoestrogens in human serum measured by whole cell uptake assays: physiology of delivery modifies estrogenic activity, Proc. Soc. Exp. Biol. Med. 217 (3) (1998) 300–309.
[78] S.C. Nagel, F.S. vom Saal, W.V. Welshons, Developmental effects of estrogenic chemicals are predicted by an in vitro assay incorporating modification of cell uptake by serum, J. Steroid Biochem. Mol. Biol. 69 (1–6) (1999) 343–357.
[79] D. Sheehan, M. Young, Diethylstilbestrol and estradiol binding to serum albumin and pregnancy plasma of rats and humans, Endocrinology 104 (1979) 1442–1446.
[80] G. Majdic, R.M. Sharpe, P.T. Saunders, Maternal oestrogen/xenoestrogen exposure alters expression of steroidogenic factor-1 (SF-1/Ad4BP) in the fetal rat testis, Mol. Cell Endocrinol. 127 (1) (1997) 91–98.
[81] N. Atanassova, C. McKinnell, M. Walker, K.J. Turner, J.S. Fisher, M. Morley, M.R. Millar, N.P. Groome, R.M. Sharpe, Permanent effects of neonatal estrogen exposure in rats on reproductive hormone levels, Sertoli cell number, and the efficiency of spermatogenesis in adulthood, Endocrinology 140 (11) (1999) 5364–5373.
[82] L. Pylkkanen, S. Makela, R. Santti, Animal models for the preneoplastic lesions of the prostate, Eur. Urol. 30 (2) (1996) 243–248.
[83] G.S. Prins, L. Birch, The developmental pattern of androgen receptor expression in rat prostate lobes is altered after neonatal exposure to estrogen, Endocrinology 136 (3) (1995) 1303–1314.
[84] J.W. Goldzieher, F.Z. Stanczyk, Oral contraceptives and individual variability of circulating levels of ethinyl estradiol and progestins, Contraception 78 (1) (2008) 4–9.
[85] NRC, Hormonally Active Agents in the Environment, National Academies Press, Washington, DC, 1999.
[86] D.J. Nonneman, V.K. Ganjam, W.V. Welshons, F.S. Vom Saal, Intrauterine position effects on steroid metabolism and steroid receptors of reproductive organs in male mice, Biol. Reprod. 47 (5) (1992) 723–729.
[87] V.M. Kumar, M.E. Leo, D.J. Tindall, Modulation of androgen receptor transcriptional activity by the estrogen receptor, J. Androl. 15 (1994) 534–542.
[88] A.T. Collins, B. Zhiming, K. Gilmore, D.E. Neal, Androgen and oestrogen responsiveness of stromal cells derived from the human hyperplastic prostate: oestrogen regulation of the androgen receptor, J. Endocrinol. 143 (1994) 269–277.
[89] A. Migliaccio, G. Castoria, M. Di Domenico, A. de Falco, A. Bilancio, M. Lombardi, M.V. Barone, D. Ametrano, M.S. Zannini, C. Abbondanza, F. Auricchio, Steroid-induced androgen receptor-oestradiol receptor b-Src complex triggers prostate cancer cell proliferation, EMBO J. 19 (20) (2000) 5406–5417.
[90] S.R. Lee, S.M. Ramos, A. Ko, D. Masiello, K.D. Swanson, M.L. Lu, S.P. Balk, AR and ER interaction with a p21-activated kinase (PAK6), Mol. Endocrinol. 16 (1) (2002) 85–99.
[91] O.O. Adesanya-Famuyiwa, J.A. Zhou, G. Wu, C. Bondy, Localization and sex steroid regulation of androgen receptor gene expression in rhesus monkey uterus, Obst. Gynecol. 93 (2) (1999) 265–270.
[92] T. Esposito, E. Astore, A. Cardone, F. Angelini, B. Varriale, Regulation of androgen receptor mRNA expression in primary culture of Harderian gland cells: cross-talk between steroid hormones, Comparative Biochem. Physiol. B: Biochem. Mol. Biol. 132 (1) (2002) 97–105.
[93] K. Suzuki, K. Ito, T. Suzuki, S. Honma, H. Yamanaka, Synergistic effects of estrogen and androgen on the prostate: effects of estrogen on androgen- and estrogen-receptors, BrdU uptake, immunohistochemical study of AR, and responses to antiandrogens, Prostate 26 (3) (1995) 151–163.
[94] J. Bektic, O.A. Wrulich, G. Dobler, K. Kofler, F. Ueberall, Z. Culig, G. Bartsch, H. Klocker, Identification of genes involved in estrogenic action in the human prostate using microarray analysis, Genomics 83 (1) (2004) 34–44.
[95] F.S. vom Saal, Sexual differentiation in litter-bearing mammals: influence of sex of adjacent fetuses in utero, J. Anim. Sci. 67 (7) (1989) 1824–1840.
[96] M.M. Montano, W.V. Welshons, F.S. vom Saal, Free estradiol in serum and brain uptake of estradiol during fetal and neonatal sexual differentiation in female rats, Biol. Reprod. 53 (5) (1995) 1198–1207.
[97] S.M. Ho, W.Y. Tang, J. Belmonte de Frausto, G.S. Prins, Developmental exposure to estradiol and bisphenol A increases susceptibility to prostate carcinogenesis and epigenetically regulates phosphodiesterase type 4 variant 4, Cancer Res. 66 (11) (2006) 5624–5632.
[98] G.S. Prins, S.H. Ye, L. Birch, S.M. Ho, K. Kannan, Serum bisphenol A pharmacokinetics and prostate neoplastic responses following oral and subcutaneous exposures in neonatal Sprague-Dawley rats, Reprod. Toxicol. 31 (1) (2011) 1–9.
[99] J. Trachtenberg, L.L. Hicks, P.C. Walsh, Androgen- and estrogen-receptor content in spontaneous and experimentally induced canine prostatic hyperplasia, J. Clin. Invest. 65 (1980) 1051–1059.
[100] C.E. Roselli, T.A. Fasasi, Estradiol increases the duration of nuclear androgen receptor occupation in the preoptic area of the male rat treated with dihydrotestosterone, J. Steroid Biochem. Mol. Biol. 42 (2) (1992) 161–168.
[101] J.R.G. Challis, S.J. Lye, Parturition, in: E. Knobil, J. Neill, D. Pfaff (Eds.), Physiology of Reproduction, Raven Press, New York, 1994, pp. 985–1031.
[102] J.H. Clark, S.K. Mani, Actions of ovarian steroid hormones, in: E. Knobil, J. Neill, D. Pfaff (Eds.), Physiology of Reproduction, Raven Press, New York, 1994, pp. 1011–1059.
[103] S.J. McPherson, H. Wang, M.E. Jones, J. Pedersen, T.P. Iismaa, N. Wreford, E.R. Simpson, G.P. Risbridger, Elevated androgens and prolactin in aromatase-deficient mice cause enlargement, but not malignancy, of the prostate gland, Endocrinology 142 (6) (2001) 2458–2467.
[104] J.P Myers, T.J. Zoeller, F.S. vom Saal, A clash of old and new scientific concepts in toxicity, with important implications for public health, Environ. Health Perspect. 117 (2009) 1652–1655.
[105] F.S. vom Saal, D.M. Sheehan, Challenging risk assessment, Forum Appl. Res. Public Policy 13 (1998) 11–18.
[106] J. Gorski, F. Gannon, Current models of steroid hormone action: a critique, Annu. Rev. Physiol. 38 (1976) 425–450.
[107] T.O. Fox, Androgen and estrogen binding macromolecules in developing mouse brain: Biochemical and genetic evidence, Proc. Natl. Acad. Sci. 72 (1975) 4303–4307.

[108] K.R. Coser, J. Chesnes, J. Hur, S. Ray, K.J. Isselbacher, T. Shioda, Global analysis of ligand sensitivity of estrogen inducible and suppressible genes in MCF7/BUS breast cancer cells by DNA microarray, Proc. Natl. Acad. Sci. U S A 100 (2003) 13994–13999.
[109] T. Shioda, J. Chesnes, K.R. Coser, L. Zou, J. Hur, K.L. Dean, C. Sonnenschein, A.M. Soto, K.J. Isselbacher, Importance of dosage standardization for interpreting transcriptomal signature profiles: evidence from studies of xenoestrogens, Proc. Natl. Acad. Sci. 103 (32) (2006) 12033–12038.
[110] C.A. Richter, J.A. Taylor, R.R. Ruhlen, W.V. Welshons, F.S. vom Saal, Estradiol and bisphenol A stimulate androgen receptor and estrogen receptor gene expression in fetal mouse prostate cells, Environ. Health Perspect. 115 (2007) 902–908.
[111] S.J. Ellem, G.P. Risbridger, Aromatase and regulating the estrogen:androgen ratio in the prostate gland, J. Steroid Biochem. Mol. Biol. 118 (4–5) (2010) 246–251.
[112] S. Arase, K. Ishii, K. Igarashi, K. Aisaki, Y. Yoshio, A. Matsushima, Y. Shimohigashi, K. Arima, J. Kanno, Y. Sugimura, Endocrine disrupter bisphenol a increases in situ estrogen production in the mouse urogenital sinus, Biol. Reprod. 84 (4) (2011) 734–742.
[113] R.L. Ruhlen, J.A. Taylor, J. Mao, J. Kirkpatrick, W.V. Welshons, F.S. vomSaal, Choice of animal feed can alter fetal steroid levels and mask developmental effects of endocrine disrupting chemicals, J. Develop. Origins Health Disease 2 (1) (2011) 36–48.
[114] S.A. Bustin, Absolute quantification of mRNA using real-time reverse transcription polymerase chain reaction assays, J. Mol. Endocrinol. 25 (2) (2000) 169–193.
[115] G.R. Cunha, H. Fujii, B.L. Neubauer, J.M. Shannon, L. Sawyer, B.A. Reese, Epithelial-mesenchymal interactions in prostatic development. I. morphological observations of prostatic induction by urogenital sinus mesenchyme in epithelium of the adult rodent urinary bladder, J. Cell Biol. 96 (6) (1983) 1662–1670.
[116] B.G. Timms, C.W. Lee, G. Aumüller, J. Seitz, Instructive induction of prostate growth and differentiation by a defined urogenital sinus mesenchyme, Microsc. Res. Technique 30 (4) (1995) 319–332.
[117] M.M. Shen, C. Abate-Shen, Roles of the Nkx3.1 homeobox gene in prostate organogenesis and carcinogenesis, Develop. Dyn. 228 (4) (2003) 767–778.
[118] M.L. Lamm, W.S. Catbagan, R.J. Laciak, D.H. Barnett, C.M. Hebner, W. Gaffield, D. Walterhouse, P. Iannaccone, W. Bushman, Sonic hedgehog activates mesenchymal Gli1 expression during prostate ductal bud formation, Develop. Biol. 249 (2) (2002) 349–366.
[119] C. Gupta, The role of estrogen receptor, androgen receptor and growth factors in diethylstilbestrol-induced programming of prostate differentiation, Urol. Res. 28 (4) (2000) 223–229.
[120] H. Zhu, M. Mazor, Y. Kawano, M.M. Walker, H.Y. Leung, K. Armstrong, J. Waxman, R.M. Kypta, Analysis of Wnt gene expression in prostate cancer: mutual inhibition by WNT11 and the androgen receptor, Cancer Res. 64 (21) (2004) 7918–7926.
[121] T. Ishitani, S. Kishida, J. Hyodo-Miura, N. Ueno, J. Yasuda, M. Waterman, H. Shibuya, R.T. Moon, J. Ninomiya-Tsuji, K. Matsumoto, The TAK1-NLK mitogen-activated protein kinase cascade functions in the Wnt-5a/Ca(2+) pathway to antagonize Wnt/beta-catenin signaling, Mol. Cell. Biol. 23 (1) (2003) 131–139.
[122] H. Shimizu, M.A. Julius, M. Giarre, Z. Zheng, A.M. Brown, J. Kitajewski, Transformation by Wnt family proteins correlates with regulation of beta-catenin, Cell Growth Differ. 8 (12) (1997) 1349–1358.
[123] C.I. Truica, S. Byers, E.P. Gelmann, Beta-catenin affects androgen receptor transcriptional activity and ligand specificity, Cancer Res. 60 (17) (2000) 4709–4713.
[124] C. Oka, R. Tsujimoto, M. Kajikawa, K. Koshiba-Takeuchi, J. Ina, M. Yano, A. Tsuchiya, Y. Ueta, A. Soma, H. Kanda, M. Matsumoto, M. Kawaichi, HtrA1 serine protease inhibits signaling mediated by Tgfbeta family proteins, Development 131 (5) (2004) 1041–1053.
[125] Z. Wang, W. Shu, M.M. Lu, E.E. Morrisey, Wnt7b activates canonical signaling in epithelial and vascular smooth muscle cells through interactions with Fzd1, Fzd10, and LRP5, Mol. Cell. Biol. 25 (12) (2005) 5022–5030.
[126] P. Bovolenta, P. Esteve, J.M. Ruiz, E. Cisneros, J. Lopez-Rios, Beyond Wnt inhibition: new functions of secreted Frizzled-related proteins in development and disease, J. Cell Sci. 121 (Pt 6) (2008) 737–746.
[127] H. Pettersson, J. Lundqvist, E. Oliw, M. Norlin, CYP7B1-mediated metabolism of 5alpha-androstane-3alpha, 17beta-diol (3alpha-Adiol): a novel pathway for potential regulation of the cellular levels of androgens and neurosteroids, Biochim. Biophys. Acta 1791 (12) (2009) 1206–1215.
[128] Z. Weihua, R. Lathe, M. Warner, J.A. Gustafsson, An endocrine pathway in the prostate, ERbeta, AR, 5alpha-androstane-3beta, 17beta-diol, and CYP7B1, regulates prostate growth, Proc. Natl. Acad. Sci. U S A 99 (21) (2002) 13589–13594.
[129] F.S. vom Saal, C. Hughes, An extensive new literature concerning low-dose effects of bisphenol A shows the need for a new risk assessment, Environ. Health Perspect. 113 (2005) 926–933.
[130] L.N. Vandenberg, M.V. Maffini, C. Sonnenschein, B.S. Rubin, A.M. Soto, Bisphenol-A and the great divide: a review of controversies in the field of endocrine disruption, Endocr. Rev. 30 (1) (2009) 75–95.
[131] S.Z. Cagen, J.M. Waechter, S.S. Dimond, W.J. Breslin, J.H. Butala, F.W. Jekat, R.L. Joiner, R.N. Shiotsuka, G.E. Veenstra, L.R. Harris, Normal reproductive organ development in CF-1 mice following prenatal exposure to Bisphenol A, Toxicol. Sci. 11 (1999) 15–29.
[132] R.W. Tyl, C. Myers, M. Marr, C.S. Sloan, N. Castillo, M.M. Veselica, J.C. Seely, S.S. Dimond, J.P. Van Miller, R.S. Shiotsuka, D. Beyer, S.G. Hentges, J.M. Waechter, Two-Generation Reproductive Toxicity Study of Dietary Bisphenol A (BPA) in CD-1® (Swiss) Mice, Toxicol. Sci. 102 (2008) 362–384.
[133] J.P. Myers, F.S. vom Saal, B.T. Akingbemi, K. Arizono, S. Belcher, T. Colborn, I. Chahoud, D.A. Crain, F. Farabollini, L.J. Guillette Jr., T. Hassold, S.M. Ho, P.A. Hunt, T. Iguchi, S. Jobling, J. Kanno, H. Laufer, M. Marcus, J.A. McLachlan, A. Nadal, J. Oehlmann, N. Olea, P. Palanza, S. Parmigiani, B.S. Rubin, G. Schoenfelder, C. Sonnenschein, A.M. Soto, C.E. Talsness, J.A. Taylor, L.N. Vandenberg, J.G. Vandenbergh, S. Vogel, C.S. Watson, W.V. Welshons, R.T. Zoeller, Why public health agencies cannot depend on good laboratory practices as a criterion for selecting data: the case of bisphenol A, Environ. Health Perspect. 117 (3) (2009) 309–315.
[134] T. Tweedale, Good laboratory practices and safety assessments: another view, Environ. Health Perspect. 118 (5) (2010) 194.
[135] C.R. Cederroth, M. Vinciguerra, F. Kühne, R. Madani, M. Klein, R.W. James, D.R. Doerge, T.J. Visser, M. Foti, F. Rohner-Jeanrenaud, J.-D. Vassalli, S. Nef, A phytoestrogen-rich diet increases energy expenditure and decreases adiposity in mice, Environ. Health Perspect. 115 (10) (2007) 1467–1473.
[136] J.J. Heindel, F.S. vom Saal, Meeting report: batch-to-batch variability in estrogenic activity in commercial animal diets—importance and approaches for laboratory animal research, Environ. Health Perspect. 116 (3) (2008) 389–393.
[137] R.L. Ruhlen, K.L. Howdeshell, J. Mao, J.A. Taylor, F.H. Bronson, R.R. Newbold, W.V. Welshons, F.S. vom Saal, Low phytoestrogen levels in feed increase fetal serum estradiol resulting in the "fetal estrogenization syndrome" and obesity in CD-1 mice, Environ. Health Perspect. 116 (3) (2008) 322–328.
[138] D.C. Dolinoy, D. Huang, R.L. Jirtle, Maternal nutrient supplementation counteracts bisphenol A-induced DNA hypomethylation in early development, Proc. Natl. Acad. Sci. U S A 104 (32) (2007) 13056–13061.
[139] K.L. Howdeshell, J. Furr, C.R. Lambright, V.S. Wilson, B.C. Ryan, L.E. Gray Jr., Gestational and lactational exposure to ethinyl estradiol, but not bisphenol A, decreases androgen-dependent reproductive organ weights and epididymal sperm abundance in the male long evans hooded rat, Toxicol. Sci. 102 (2) (2008) 371–382.
[140] R.W. Tyl, Basic exploratory research versus guideline-compliant studies used for hazard evaluation and risk assessment: bisphenol A as a case study, Environ. Health Perspect. 117 (11) (2009) 1644–1651.
[141] EFSA, Opinion of the Scientific Panel on Food Additives, Flavourings, Processing Aids and Materials in Contact with Food on a request from the Commission related to 2,2-bis(4-hydroxyphenyl)propane (Bisphenol A), The EFSA (European Food Safety Authority) J. 428 (2006) 1–76.
[142] IRIS, Bisphenol A. (CASRN 80-05-7); US-EPA Integrated Risk Information System Substance file; http://www.epa.gov/iris/subst/0356.htm. Access date: November 25, 2007, 1988.
[143] F. Reyes, R. Boroditsky, J. Winter, C. Faiman, Studies on human sexual development. II. Fetal and maternal serum gonadotropin and sex steroid concentrations, J. Clin. Endocrinol. Metab. 38 (1974) 612–617.
[144] J.J. Heindel, R.E. Chapin, J. George, D.K. Gulati, P.A. Fail, L.H. Barnes, R.S. Yang, Assessment of the reproductive toxicity of a complex mixture of 25 groundwater contaminants in mice and rats, Fundam. Appl. Toxicol. 25 (1) (1995) 9–19.
[145] D.K. Li, J.R. Daling, B.A. Mueller, D.E. Hickok, A.G. Fantel, N.S. Weiss, Oral contraceptive use after conception in relation to the risk of congenital urinary tract anomalies, Teratology 51 (1) (1995) 30–36.
[146] K.A. Thayer, R.L. Ruhlen, K.L. Howdeshell, D.L. Buchanan, P.S. Cooke, D. Preziosi, W.V. Welshons, J. Haseman, F.S. vom Saal, Altered prostate growth and daily sperm production in male mice exposed prenatally to subclinical doses of 17alpha-ethinyl oestradiol, Hum. Reprod. 16 (5) (2001) 988–996.
[147] C.L. Ogden, M.D. Carroll, L.R. Curtin, M.A. McDowell, C.J. Tabak, K.M. Flegal, Prevalence of overweight and obesity in the United States, 1999-2004, JAMA 295 (13) (2006) 1549–1555.
[148] W.V. Welshons, F.S. vom Saal, The importance pf protocol design and data reporting to research on endocrine disruption: response, Environ. Health Perspect. 106 (1998) A316–A317.
[149] S.G. Driscoll, S.H. Taylor, Effects of prenatal maternal estrogen on the male urogenital system, Obst. Gynecol. 56 (5) (1980) 537–542.
[150] A. Bjornerem, B. Straume, M. Midtby, V. Fonnebo, J. Sundsfjord, J. Svartberg, G. Acharya, P. Oian, G.K. Berntsen, Endogenous sex hormones in relation to age, sex, lifestyle factors, and chronic diseases in a general population: the Tromso Study, J. Clin. Endocrinol. Metab. 89 (12) (2004) 6039–6047.
